# Effect of molybdenum and tungsten on the reduction of nitrate in nitrate reductase, a DFT study

**DOI:** 10.1186/s13065-017-0263-7

**Published:** 2017-04-26

**Authors:** Uzma Habib, Matthias Hoffman

**Affiliations:** 1 Research Center for Modeling and Simulation (RCMS), National University of Science and Technology (NUST), H-12, Islamabad, Pakistan; 2 Institute of Inorganic Chemistry, Heidelberg University, Heidelberg, Germany

**Keywords:** Nitrate reductase, DFT studies, Molybdenum, Tungsten

## Abstract

The molybdenum and tungsten active site model complexes, derived from the protein X-ray crystal structure of the first W-containing nitrate reductase isolated from *Pyrobaculum aerophilum*, were computed for nitrate reduction at the COSMO-B3LYP/SDDp//B3LYP/Lanl2DZ(p) energy level of density functional theory. The molybdenum containing active site model complex (Mo–Nar) has the largest activation energy (34.4 kcal/mol) for the oxygen atom transfer from the nitrate to the metal center as compared to the tungsten containing active site model complex (W–Nar) (12.0 kcal/mol). Oxidation of the educt complex is close to thermoneutral (−1.9 kcal/mol) for the Mo active site model complex but strongly exothermic (−34.7 kcal/mol) for the W containing active site model complex, however, the M^VI^ to M^IV^ reduction requires equal amount of reductive power for both metal complexes, Mo–Nar or W–Nar.

**Electronic supplementary material:**

The online version of this article (doi:10.1186/s13065-017-0263-7) contains supplementary material, which is available to authorized users.

## Background

Molybdenum and tungsten are the only 4d (Mo) and 5d (W) transition metals prefer to be essential for biological systems. Mononuclear enzymes containing Mo or W at their active sites generally catalyze oxygen atom transfer reactions [1, 2]. Despite the high similarity between the chemical properties of Mo and W, W-containing enzymes are by far less common. Mo-containing enzymes are found in almost all forms of life [1], whereas W-containing enzymes seem to be popular for organisms such as hyperthermophilic archaea that live in extreme environments [2]. However, W-containing enzymes have also been found in organisms that do not need extreme conditions [3–5], suggesting a more important role for tungsten [6].

**Fig. 1:**
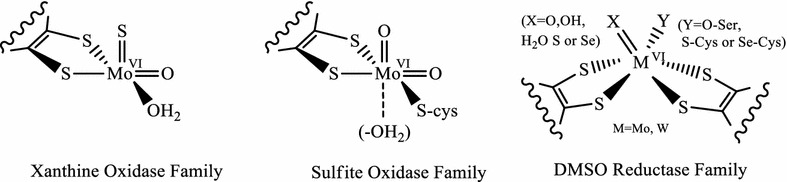
Active site composition of subfamilies of mononuclear Mo/W enzymes

Mononuclear enzymes contain a cofactor that comprises metallopterin (MPT) or some of its nucleotide variants, each of which is coordinated to Mo or W with an enedithiolene motif. Based on the active site structure and type of reaction they catalyze, these mononuclear MPT containing enzymes have been grouped into three subfamilies (Fig. 1), xanthine oxidase family, sulfite oxidase family, and DMSO (dimethylsulfoxide) reductase family [1].

Nitrate reductases (NRs) play key roles in the first step of biological nitrogen cycles [7–9] i.e., assimilatory ammonification (to incorporate nitrogen into biomolecules), denitrification (to generate energy for cellular function) and dissimilatory ammonification (to dissipate extra energy by respiration). They always catalyze the reduction of nitrate to nitrite, and have been classified into three groups, assimilatory nitrate reductases (Nas), respiratory nitrate reductases (Nar) and periplasmic nitrate reductases (Nap). Nas belongs to the sulfite oxidase family and is located in the cytoplasm [10]. It is the first enzyme of a reduction sequence for nitrogen incorporation into the biomass that maintains the bioavailability of nitrate to plants, algae, fungi, archaea and bacteria [11, 12]. Dissimilatory nitrate reductases, Nar and Nap belong to the DMSO reductase family of mononuclear MPT containing molybdo-enzymes. They are linked to respiratory electron transport systems and are located in the membrane and periplasm, respectively. They catalyze the first step of the catabolic, anaerobic respiration pathway in bacteria and archaea [14].

Nitrate reduction, catalyzed by membrane bound respiratory nitrate reductase (Nar), is an important step of the denitrification in the anaerobic respiratory pathways employed by a diverse group of bacteria and archaea [13]. Nar was found to contain a Mo cofactor in all microbes from which it was isolated and belongs to the DMSO reductase family [14]. In general, Nar becomes inactive by the addition of tungstate (WO_4_^2−^) to the growth medium [15], although due to similar chemical properties W can replace Mo as the active site metal and cannot only retain but increase its catalytic activity in *E. coli* TMAO reductase [16], the *Desulfovibrio alaskensis* formate dehydrogenase [17] and the *Rhodobactercapsulatus* DMSO reductase [18]. However, recently the nitrate reductase (Nar) from the hyperthermophilic denitrifying archaeon *Pyrobaculum aerophilum* has been shown to retain its activity even at a tungsten rich environment [19].

*Pyrobaculum aerophilum*, a hyperthermophilic archaeon, is naturally exposed to high levels of tungsten, a heavy metal that is abundant in high temperature environments. Tungsten was reported to stimulate the growth of several mesophilic methanogens and some mesophilic and thermophilic bacteria [14]. The growth of *P. aerophilum* also depends on the presence of tungstate in the growth medium which suggests the involvement of tungstoenzymes in essential metabolic pathways [20].

*Pyrobaculum aerophilum* is the only hyperthermophilic archaeon isolated that reduces nitrate via a membrane bound respiratory nitrate reductase (Nar) [20]. Nar purified from *P. aerophilum* grown in the absence of added molybdate (MoO_4_^2−^) and with 4.5 µM tungstate (WO_4_^2−^) is a tungsten containing enzyme, which is identical to Mo-Nar [21] (previously isolated from *P. aerophilum*), indicating that either metal can serve as the active site ion. The crystal structure is similar to the previously reported Nar from *E. coli* [22], a heterodimeric enzyme termed as NarGH where NarG hosts the metal (Mo or W) catalytic site. The metal is coordinated by two metallopterin guanine dinucleotide (*bis*-MGD) ligands, a carboxyl group of Asp_222_ and a water molecule. The NarH component possesses an iron–sulfur (FeS) redox active subunit [19].

NarGH reduces nitrate to nitrite, changing the oxidation state of metal from +IV to +VI. Two electrons and two protons are required for the reductive half reaction, resulting in the formation of a water molecule and a nitrite ion (Eq. 1).

1 $${\text{NO}}_{ 3}^{ - } + {\text{ 2H}}^{ + } + {\text{ 2e}}^{ - } \rightleftharpoons {\text{NO}}_{ 2}^{ - } + {\text{ H}}_{ 2} {\text{O }}.$$

**Fig. 2:**
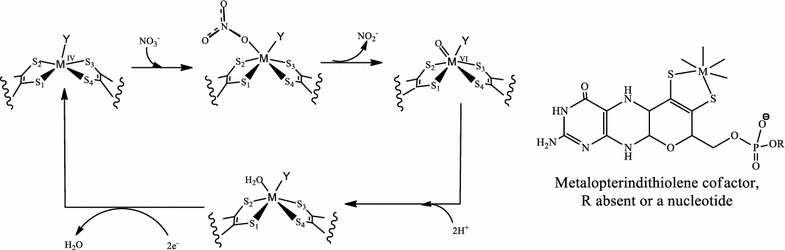
Schematic description of the proposed mechansim [1] for the nitrate reduction, where M=Mo and Y=S–Cys. Also the metalopterin dinucleotide cofactor is shown

The active site of dissimilatory nitrate reductase (*Desulfovibrio desulfuricans*), in the reduced state contains a Mo atom bound by two metalopterin dithiolene ligands and a cysteinate residue. An experimental study on small model complexes demonstrates that nitrate reduction by primary (direct) oxo transfer [23] is a feasible reaction pathway (Fig. 2) [24].

Here we present a density functional theory (DFT) study on model complexes derived from the protein X-ray crystal structure of *P. aerophilum* [19] nitrate reductase (Nar). The purpose of the study was to investigate (i) the effect on the reduction of nitrate when W replaces Mo at the active site, (ii) the energy barriers on the potential energy surface and (iii) the reason for the activity loss of Nars (respiratory nitrate reductase) in the presence of W.

## Computational details

All geometries were optimized using Gaussian 09 with the hybrid density functional B3LYP [25] and the LANL2DZ basis set [26–29] augmented by polarization functions on sulfur atoms (ζ = 0.421) [30]. The starting nitrate complex geometries for transition state searches were generated by shortening and lengthening of forming and breaking bonds, respectively. Frequency calculations proved transition states to have exactly one imaginary frequency with the correct transition vector. Single point energies were computed with the B3LYP functional and the Stuttgart–Dresden effective core potential basis set (SDD) [31, 32] augmented by polarization functions for all atoms except Mo, W and H (ζ = 0.600, 1.154, 0.864, and 0.421 for C, O, N, and S, respectively) [30]. Self-consistent reaction field (SCRF) computations were performed on the optimized geometries to model the protein surrounding the active site by a conductor like polarizable continuum method (CPCM) [33] as implemented in Gaussian 09 [34, 35]. Default Gaussian 03 parameters were used for the evaluation of solute–solvent dispersion and repulsion interaction energies [36, 37], and solute cavitation energy variations [38]. The molecular cavity was specified using a minimum radius (RMin) of 0.5Ǻ and an overlap index (OFac) of 0.8 [39].

## Active site models

**Fig. 3:**
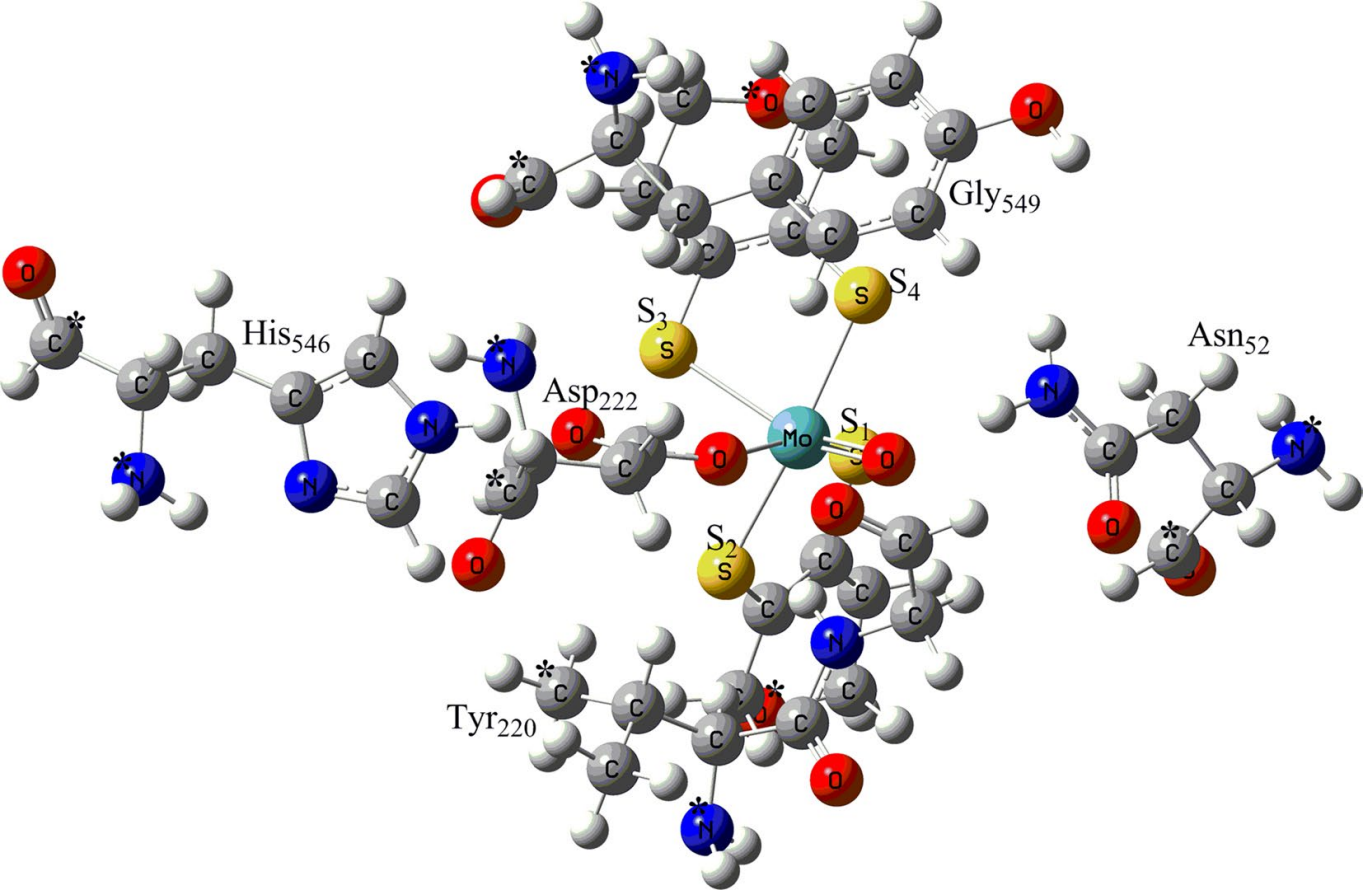
Optimized oxidized active site model of Mo-Nar. Atoms labeled (*) were kept fixed at their X-ray crystal structure positions

Two types of active site models were designed on the basis of the protein X-ray crystal structure of *P. aerophilum* (PDB ID: 1R27) [19] only differing in the metal center, **a** containing Mo and **b** containing W at the active site. These active site models include the metal center coordinated by two enedithiolene moieties of the pterin molecules, by Asp_222_ and by H_2_O_8538_. His_546_, Asn_52_, Tyr_220_, Gly_549_ and Val_578_ residues were also included in the model complexes as they may influence the catalytic reaction due to their proximity to the metal center. Hydrogen atoms were added manually. His_546_ and Gly_549_ residues form hydrogen bonds to the ionized Asp_222_ preventing it to rotate and become a bidentate ligand which then would block the substrate binding site. Asn_52_ was included as its distance of 3.9 Ǻ from the metal center suggests that it is suitable for substrate coordination [19]. During the optimizations, alpha (α) carbon atoms and nitrogen atoms attached to the beta (β) carbon atoms of His_546_, Asn_52_, Tyr_220_ and Asp_222_ were kept fixed to their crystal structure positions to mimic the steric constraints by the protein matrix. Carbon atom C_7_ and the nitrogen atom attached to carbon atom C_5_ were kept fixed for residue Gly_549_. The MPT ligands were truncated at the pyran rings and oxygen atoms of these pyran rings were also kept fixed (Fig. 3).

First, hydrogen atoms were geometry optimized applying one negative overall charge (assuming Mo/W at the +VI oxidation state), keeping all heavy-atoms fixed at their positions. The resulting geometries served to generate the different starting geometries needed for computing the mechanism for nitrate reduction.

The starting geometries for the substrate and product complexes are generated by slight distortion of M–O and O–NO_2_ in the optimized transition state geometries, **6a** and **6b**. Geometries with slightly elongated M–O distance and reduced O–NO_2_ distance are considered as the starting geometries for the optimization of **5a** and **5b** educt-substrate complexes whereas reduced M–O distance and elongated O–NO_2_ distance are considered as the starting geometries for the optimization of **7a** and **7b** product complexes. The geometry optimizations of these distorted geometries directly lead to complexes, **5a**/**5b** and **7a**/**7b**.

## Results

### Optimized active site model complexes

The protein X-ray crystal structure of *P. aerophilum* Nar from the PDB data base (PDB ID: 1R27) [19] shows that at the active site the metal is coordinated by two metallopterin guanine dinucleotide (*bis*-MGD) ligands, a carboxyl group of Asp_222_ and a water molecule [19]. However, the distance of the oxygen atom (O_wat_) of this coordinated water molecule from the metal center is 1.87 Ǻ which neither falls in the range expected for metal oxide (1.71–1.75 Ǻ) [40, 41], nor for water (2.0–2.3 Ǻ) [42] ligands. Also, the distance between O_wat_ and oxygen of Asp_222_ (O_Asp_) is 1.59 Ǻ, which is only 0.1 Ǻ longer than the typical peroxo O–O^−^ bond length (1.49 Ǻ).

**Fig. 4:**
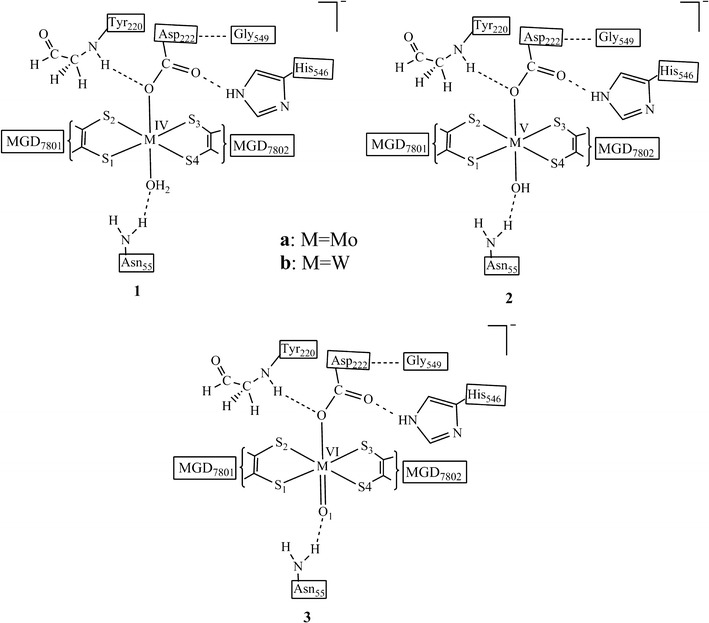
The chemical structure of the active site model complexes **1** and **2** derived from the protein X-ray crystal structure of Nar (PDB ID 1R27) [19]

We have optimized three active site model complexes to clarify the nature of this oxo species; **1** (oxidation state of Mo/W is +IV, overall charge is −1) contains a water molecule, **2** (oxidation state of Mo/W is +V, overall charge is −1) contains a hydroxide ligand and **3** (oxidation state of Mo/W is +VI, overall charge is −1) contains an oxide (O_1_) group attached to the metal (Fig. 4).

**Fig. 5:**
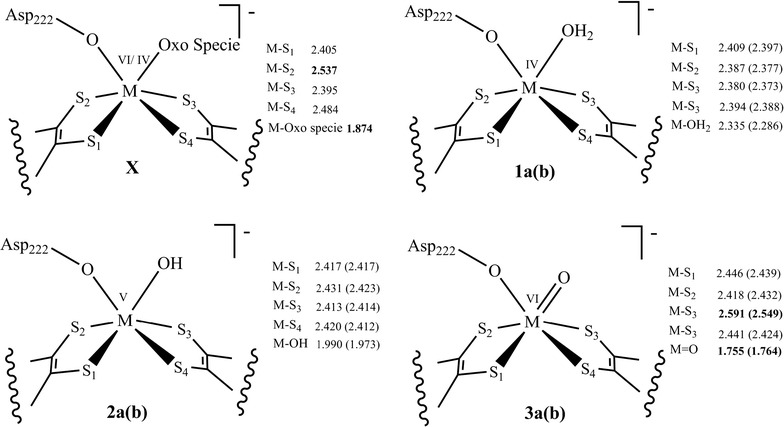
The chemical structures of the active site model complex of protein X-ray crystal structure of Nar (PDB ID 1R27) [19] represented as** X** as well as the active site model complexes derived from the protein X-ray crystal structure of Nar (PDB ID 1R27) [19] showing metal–sulfur and metal–oxo specie bond distances. Where, model **1** represents the presence of M–OH_2_ bond, **2** represents the presence of M–OH bond, **3** represents the presence of M=O bond, however, **a** and **b** represents the Mo and W, respectively, as the metal at the active site

Geometry optimizations of active site model complexes **1**, **2** and **3** results in distinctively different geometrical parameters of the metal coordination site relative to the protein X-ray crystal structure geometry of NarGH [19]. Optimized geometry data for the model complexes **1a** with M=Mo (**1b**, M=W) show that the dithiolenes are twisted less against each other as the S_1_–S_2_–S_3_–S_4_ dihedral angle decreases from −18.3° to −6.4° for **1a** (−2.5° for **1b**) i.e., the coordination geometries are nearly trigonal prismatic (Tables 1, 2). Bond distances between the metal center, M and the dithiolene sulfur atoms decreases from ~2.455 to ~2.393 Ǻ (~2.384 Ǻ) when comparison is made with the protein X-ray crystal structure (Fig. 5; Tables 1, 2). Elongated bond distances for M–O_wat_ [from 1.874 to 2.335 Ǻ (2.286 Ǻ)] and M–O_Asp_ [from 1.97 to 2.142 Ǻ (2.122 Ǻ)] are computed. But the main difference lies in the Mo–S_2_ bond distance (from 2.537 to 2.387 Ǻ) (Fig. 5; Tables 1, 2), in the bond angles between the O_Asp_, M and O_wat_ [from 49° to 66° (66°)], and in the distance between the two oxygen atoms, O_Asp_–O_wat_ [from 1.596 to 2.428 Ǻ (2.392Ǻ)].

**Table 1:** Geometrical features of the optimized model complexes of the reaction mechanism for the molybdenum containing nitrate reductase

	Crystal structure	Reduced complex^a^ **1a**	Oxidized complex^b^ **2a**	Oxidized complex^c^ **3a**	Reduced complex **4a**	Educt complex **5a**	Transition state **6a**	Product complex **7a**
Mo–S_1_ (Ǻ)	2.405	2.409	2.417	2.446	2.379	2.370	2.420	2.430
Mo–S_2_ (Ǻ)	2.537	2.387	2.431	2.418	2.347	2.348	2.452	2.629
Mo–S_3_ (Ǻ)	2.395	2.380	2.413	2.591	2.345	2.349	2.422	2.421
Mo–S_4_ (Ǻ)	2.484	2.394	2.420	2.441	2.375	2.371	2.457	2.475
Mo–O_Asp_ (Ǻ)	1.97	2.142	2.145	2.083	2.017	2.029	2.102	2.133
Mo–O_wat/OH/O1_ (Ǻ)	1.874	2.335	1.990	1.755	–	–	–	–
Mo–O (Ǻ)	–	–	–	–	–	–	1.918	1.737
O–NO_2_^−^ (Ǻ)	–	–	–	–	–	1.310	1.723	–
O_Asp_–O_wat/1_ (Ǻ)	1.596	2.428	2.458	2.684	–	–	–	2.786
O_Asp_–Mo–O_wat/1_(°)	49.0	65.5	72.8	88.3	–	–	–	91.5
S_1_–S_2_–S_3_–S_4_(°)	−18.3	−6.4	15.1	−43.7	−0.2	2.0	30.5	54.5

^a^Water containing reduced complex

^b^Hydroxide containing oxidized complex

^c^Oxygen containing oxidized complex

**Table 2:** Geometrical features of the optimized model complexes of the reaction mechanism for the tungsten containing nitrate reductase

	Crystal structure	Reduced complex^a^ **1b**	Oxidized complex^b^ **2b**	Oxidized complex^c^ **3b**	Reduced complex **4b**	Educt complex **5b**	Transition state **6b**	Product complex **7b**
W–S_1_ (Ǻ)	2.405	2.397	2.417	2.439	2.369	2.363	2.428	2.455
W–S_2_ (Ǻ)	2.537	2.377	2.423	2.432	2.334	2.335	2.419	2.442
W–S_3_ (Ǻ)	2.395	2.373	2.414	2.549	2.337	2.337	2.424	2.562
W–S_4_ (Ǻ)	2.484	2.388	2.412	2.424	2.371	2.369	2.457	2.428
W–O_Asp_ (Ǻ)	1.97	2.122	2.113	2.040	1.980	1.986	2.079	2.076
W–O_wat/OH/O1_ (Ǻ)	1.874	2.286	1.973	1.764	–	–	–	–
W–O (Ǻ)	–	–	–	–	–	–	1.942	1.757
O–NO_2_^−^ (Ǻ)	–	–	–	–	–	1.310	1.638	–
O_Asp_–O_wat/1_ (Ǻ)	1.596	2.392	2.439	2.647	–	–	–	2.747
O_Asp_–W–O_wat/OH/1_(°)	49.0	65.6	73.2	87.9	–	–	–	91.2
S_1_–S_2_–S_3_–S_4_(°)	−18.3	−6.3	20.2	−42.1	1.3	1.2	7.6	−42.4

^a^Water containing reduced complex

^b^Hydroxide containing oxidized complex

^c^Oxygen containing oxidized complex

Distorted trigonal prismatic geometries result from geometry optimizations of oxidized model complexes **2a** (**2b**). Optimized data show changes in the S_1_–S_2_–S_3_–S_4_ dihedral angles from −18.3° to 15.1° (20.2°) and in the M–S bond distances from ~2.455 to ~2.420 Ǻ (~ 2.417 Ǻ) as compared to the protein X-ray crystal structure (Fig. 5; Tables 1, 2). Bond distances between M–O_Asp_ and M–OH are increased from 1.97 to 2.145 Ǻ (2.113 Ǻ) and from 1.874 to 1.990 Ǻ (1.973 Ǻ), respectively. O_Asp_–O_OH_ bond distance is increased from 1.596 to 2.458 Ǻ (2.439 Ǻ) and the bond angle between O_Asp_, M and O_OH_ is increased from 49° to 72.8° (73.2°).

Distorted octahedral coordination geometries result from geometry optimizations of oxidized model complexes **3a** (**3b**).Optimized data shows increase in the S_1_–S_2_–S_3_–S_4_ dihedral angles [from −18.3° to −43.7° (−42.1°)] and in the M–S bond distances [from ~2.455 to ~2.474 Ǻ (~2.461 Ǻ)]. One M–S bond is significantly longer than the other three. However, it is the M–S_2_ bond (2.537 Ǻ) in the X-ray structure while it is the M–S_3_ bond [2.591 Ǻ (2.549)] (Fig. 5; Tables 1, 2) in the optimized oxidized model complexes. These sulfur atoms (S_2_ and S_3_, respectively) are at the *trans* position relative to the oxo ligand, a trans-influencing ligand which causes the elongation of the M–S bonds.

Increased bond angles between the O_Asp_, M and O_1_ [from 49° to 88° (88°)], and distances between the two oxygen atoms, O_Asp_–O_1_ [from 1.596 to 2.684 Ǻ (2.647Ǻ)] are computed in complexes **3a** (**3b**). Slightly elongated M–O_Asp_ distances [from 1.97 to 2.083Ǻ (2.04Ǻ)] and shortened M–O_1_ distances [from 1.874 to 1.755 Ǻ (1.764Ǻ)] are also observed (Fig. 5; Tables 1, 2).

**Fig. 6:**
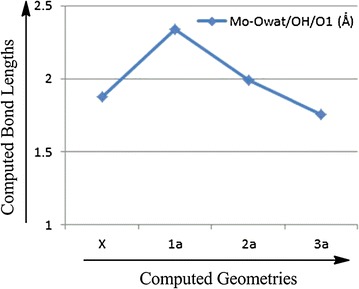
Plot of crystallographic and computed metal–oxo species bond distances, where *X* represents the experimental data and *1a*, *2a*, *3a* represents the calculated data

**Fig. 7:**
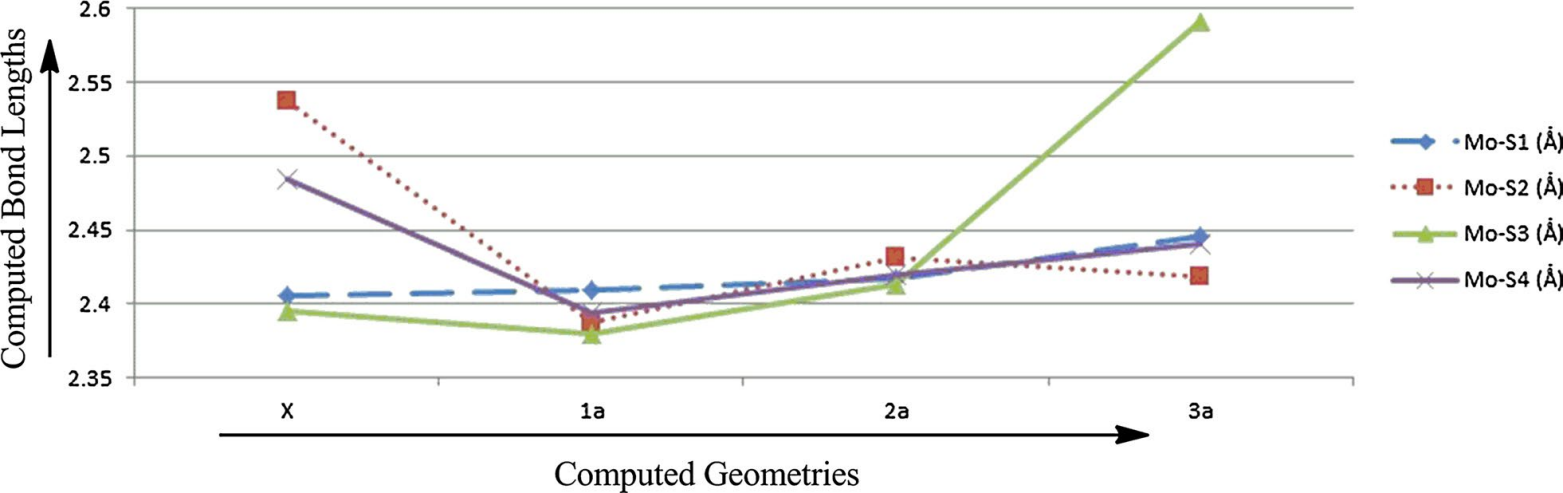
Plot of crystallographic and computed metal–sulphur bond distances where *X* represents the experimental data and *1a*, *2a*, *3a* represents the calculated data

**Fig. 8:**
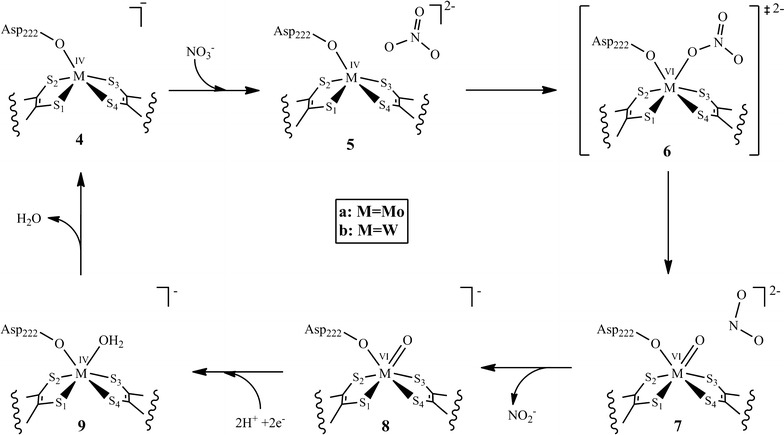
Schematic description of the mechanism for nitrate reduction at the NR active site

Comparing results from the computed model complexes **1**, **2**, **3** and the protein X-ray crystal structure, it is observed that energetically there is no difference between them, however, the M–O_1_ [1.755 Ǻ (1.764 Ǻ)] and M–OH [1.990 Ǻ (1.973 Ǻ)] bond distances in model complexes **2** and **3**, respectively, are similar to the metal oxo bond distance in X-ray crystal structure (1.874 Ǻ) (Tables 1, 2). Based on the M–O bond distance, the controversial oxo specie could most probably be the *oxide* group or *hydroxide* group. But when we compare the bond distances between metal center M and S of the dithiolenes, one M–S bond is significantly longer than the other three in optimized model complexes **3** as well as in the protein X-ray crystal structure (Figs. 6, 7; Tables 1, 2). The elongation of one M–S bond distance is due to the presence of high electronegative oxide group, in comparison to the sulfides, hydroxide and water molecules. Due to high electronegativity, shared electrons are attracted to the oxygen, resulting in a shift of electron density toward the oxide group, decreasing M–O and increasing the M–S bond distance. So, according to the computed results, this oxo specie is *oxide* (Fig. 8).

### Optimized reduced complexes 4a/4b

The reaction catalyzed by nitrate reductase is an oxo-transfer reaction, in which an oxygen atom is transferred from nitrate to the reduced metal. As a consequence of the metal reduction from M^VI^ to M^IV^, the oxo group of the oxidized M^VI^ is lost as hydroxo/water after proton uptake. Optimizations of the reduced active site model complexes **4a** (**4b**) without any additional ligand, i.e. fivefold coordinate metal center give S_1_–S_2_–S_3_–S_4_ dihedral angles of −0.2° (1.3°), resulting in nearly tetragonal pyramidal geometries. The bond distances between the metal center M and S of the dithiolenes are reduced (Tables 1, 2). The M–O_Asp_ distance is reduced to 2.017 Ǻ (1.980 Ǻ).

### Optimized substrate complexes 5a/5b

First, nitrate gets loosely bound in the active site pocket by weak interactions with the active site residues Asn_52_ and Gly_549_ resulting in the substrate complexes **5a** (**5b**) (Fig. 5). The computed reaction energies for the substrate complex formation are exothermic, −9.6 kcal/mol (−7.6 kcal/mol) in the gas phase and −4.6 kcal/mol (0.2 kcal/mol) for the polarizable continuum model. There is no significant change in geometrical parameters of the active site relative to the reduced complexes **4a** (**4b**) (Tables 1, 2).

### Optimized transition state complexes 6a/6b

Reduction of nitrate is a single step reaction in which the transfer of an oxygen atom proceeds through transition state **6a** (**6b**).The energy barrier computed for **6a**, 34.4 kcal/mol in the gas phase and 32.1 kcal/mol in the continuum, is almost three times as large as compared to that of **6b**, 12.0 kcal/mol in the gas phase and 11.0 kcal/mol in the continuum. There is also a remarkable difference in the geometries. The Mo containing transition state (**6a**) has a distorted octahedral geometry with an S_1_–S_2_–S_3_–S_4_ dihedral angle of 30.5° and Mo–S bond lengths increased from ~2.37 to ~2.45 Ǻ (Table 1). Mo–O and O–NO_2_ distances are 1.918 and 1.723 Ǻ, respectively. The Mo–O_Asp_ bond distance is elongated to 2.102 Ǻ.

The W containing transition state (**6b**) on the other hand has a distorted trigonal prismatic geometry where the S_1_–S_2_–S_3_–S_4_ dihedral angle is 7.6°. The W–S bond lengths are increased from ~2.37 to ~2.45 Ǻ (Table 2). The W–O and O–NO_2_ bond distances are 1.942 and 1.638Ǻ, respectively i.e., **6b** can be considered to be an earlier transition state than **6a**. The W–O_Asp_ distance is elongated to 2.079 Ǻ.

In the optimized geometries **6a** and **6b**, NO_3_^−^ is coordinated to the metal at the active center and also forms a hydrogen bond to the Asn_55_.

### Optimized product complexes 7a/7b

The nitrate reduction results in metal oxo product complexes **7a** (**7b**), having distorted octahedral geometries. In the optimized geometries, **7a** and **7b**, NO_2_^−^ is loosely bound in the active site pocket and make hydrogen bonds with the active site residues Asn_52_ and Gly_549_. Oxygen atom transfer is computed to be a slightly exothermic step for M=Mo where the product complex (**7a**) has a relative energy of −7.6 kcal/mol in the gas phase and −1.9 kcal/mol in the continuum. The Mo–O bond distance is reduced to 1.737 Ǻ while the O–NO_2_^−^ bond is broken (4.444 Ǻ). The S_1_–S_2_–S_3_–S_4_ dihedral angle is further increased to 54.5°, the Mo–S bond distances are also increased to ~2.629 Ǻ (Table 1). The Mo–O_Asp_ bond distance is further increased to 2.133 Ǻ.

On the contrary, the W containing product complex (**7b**) is highly exothermic, with computed relative energies of −43.3 kcal/mol in the gas phase and −34.7 kcal/mol in the continuum. The W–O bond distance is reduced to 1.757 Ǻ while the O−NO_2_^−^ bond is broken (5.133 Ǻ). The S_1_–S_2_–S_3_–S_4_ dihedral angle of the dithiolenes is decreased to −42.4°, whereas the W-S bond distances are increased to ~2.562 Ǻ (Table 2). There is no significant change in the W–O_Asp_ bond distance (2.079 instead of 2.076 Ǻ).

## Discussion

To date, few archaeal Nars have been characterized from *P. aerophilum* [21], *Haloarcula marimortui* [43, 44] and *Haloferax mediterranei* [45]. These archaeal Nars contain Mo cofactors at their active sites. It is not clear how these microbes maintain their ability to respire with nitrate using Mo-containing Nar in a high temperature environment that is naturally enriched with W but depleted of molybdate (MoO_4_^2−^) [46]. Early attempts to substitute tungsten for molybdenum in molybdo-enzymes failed because the organism was incapable of growing on the tungstate-containing medium [8]. However, the hyperthermophile *P. aerophilum* is a denitrifying archaeon requiring tungstate (WO_4_^2−^) for growth although it’s Nar is a Mo cofactor containing enzyme [20]. Afshar et al. [20] demonstrated that the external tungstate concentration affects the denitrification pathway efficiency of this archaeon, resulting in the complete denitrification only at high tungstate concentration.

Recently, Nar purified from *P. aerophilum* grown in the absence of added molybdate and with 4.5 µM tungstate has been reported [13] which is a W containing enzyme. *P. aerophilum* Nar is the first active nitrate reductase that contains a W cofactor. The presence of a W cofactor may be reflective of high concentrations of this metal at high temperatures [40]. As previously described this enzyme can also accommodate Mo as the active site metal [21].

To compare the properties of Mo and W cofactors containing enzymes, DFT calculations were performed on the active site model complexes derived from the protein X-ray crystal structure of *P. aerophilum* [19]. The crystal data shows that at the active site the metal is coordinated by *bis*-MGD ligands, a carboxyl group of Asp_222_ and an oxo specie. However, there is a controversy about the nature of oxo specie. Based on the optimized data from computed model complexes **1**, **2**, and **3**, this oxo specie is most probably the *oxide* group.

The mechanism of nitrate reduction was also investigated using DFT calculations on active site model complexes containing Mo and W at the metal center. Nitrate reduction is an oxo-transfer reaction in which nitrate is reduced to nitrite and metal is oxidized from +IV oxidation state to +VI. The mechanism starts with the substrate binding with the metal center (Mo and W) followed by oxygen atom transfer. According to the computed results, the computed energy barrier for the oxygen atom transfer from the nitrate to the metal center is 34.4 kcal/mol for the Mo active site model complex, about triple the energy barrier of the W active site model complex (12.0 kcal/mol) (Table 3). Thus, as compared to Mo–Nar, W–Nar should be more active, which is in contrast to experimental findings [13]. However, the W-substituted DMSO reductase from the *R. capsulatus* was reported to be 17 times more active in the reduction of DMSO than the Mo-substituted enzyme [16, 18, 21], but, the W-substituted DMSO reductase was inactive for the oxidation of dimethysulfide (DMS) [46].

**Table 3:** Computed energies (kcal/mol) relative to the educt–substrate complex for the nitrate reduction

	Educt complex **4**	Substrate complex **5**	Transition state complex **6**	Product complex **7**	Oxidized product without nitrite **3**	Reduced product with water **1**	Reduced product **4**	
M=Mo	0.0	−9.7	30.2	−11.6	−36.0	−151.5	−142.0	//B3LYP^a^
	0.0	−9.6	34.4	−7.6	−49.1	−141.0	−125.7	SDD^b^
	0.0	−4.6	32.1	−1.9	2.7	−147.8	−140.0	COSMO^c^
M=W	0.0	−7.8	7.0	−52.6	−36.3	−150.3	−142.0	//B3LYP^a^
	0.0	−7.6	12.0	−43.3	−27.7	−139.1	−125.7	SDD^b^
	0.0	0.2	11.0	−34.7	−28.4	−144.3	−140.0	COSMO^c^

^a^B3LYP/Lanl2DZ(p)

^b^B3LYP/SDDp//B3LYP/Lanl2DZ (p)

^c^COSMO-B3LYP/SDDp//B3LYP/Lanl2DZ(p) (see “Computational details”)

**Fig. 9:**
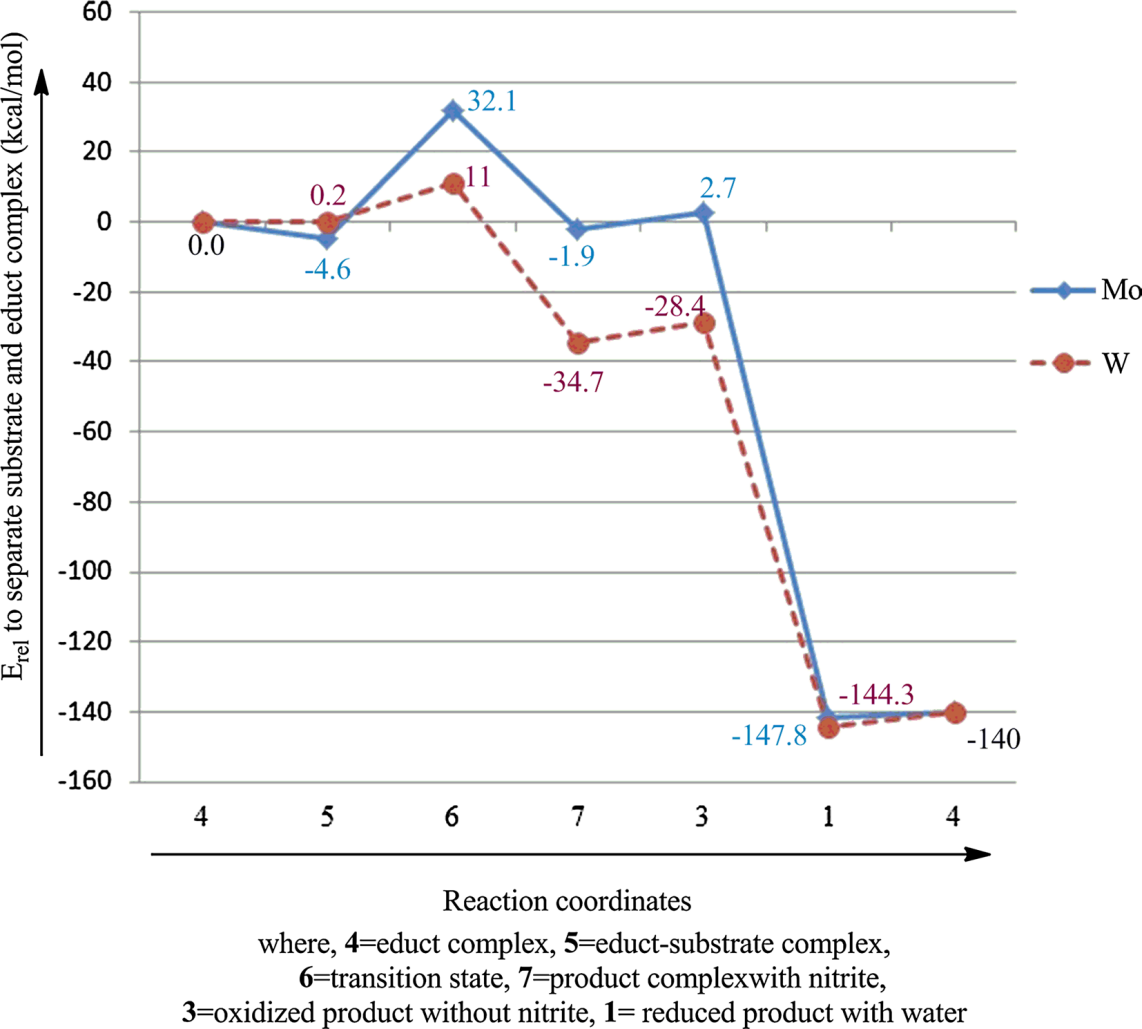
Plot of computed reaction energies (kcal/mol) relative to educt complex vs steps involved in the reaction mechanism

Oxidation of the educt complex is close to thermoneutral for the Mo active site model complex (−1.9 kcal/mol) but strongly exothermic for the W containing active site model complex (−34.7 kcal/mol) (Table 3). It was anticipated that the low relative energy for the oxidized W metal complex makes the regeneration of the +IV oxidation state much more difficult as compared to the Mo metal complex, however, calculated results shows that M^VI^ to M^IV^ reduction for both Mo and W containing metal complexes requires equal amount to reductive power i.e., 140 kcal/mol. So, although the reduction of nitrate is stimulated when W replaces Mo in the active site of Nar both the Mo containing Nar and W containing Nar requires the strong biochemical reducer (Fig. 9).

These results are in good agreement with the following experimental findings; (a) the hyperthermophile *P. aerophilum* is well adapted to a high-tungsten environment and this heavy metal is very important for its anaerobic growth mode on nitrate [21]. (b) In contrast to other mesophilic nitrate reducers, *P. aerophilum* growth with nitrate is not reduced/stopped at high tungstate concentrations [21]. Similar behaviour have been reported for NAD-dependent glutamate dehydrogenase enzyme in which enzyme isolated form hyperthermophiles shows comparable specific activities to those of enzymes from their mesophilic counterparts [47].

In conclusion, the computational result shows that the oxo specie attached with the metal at the active site of Nar is probably the *oxide* group. It is also concluded that the replacement of W with the Mo at the active site impart no effect on the overall reduction of nitrate except the energy barrier for oxygen transfer from nitrate which is low for W containing Nar (W–Nar). The most appropriate justification for this behavior of W–Nar is that *P. aerophilum* needs to support its growth by nitrate respiration even when the tungsten concentration in the environment is high; the same was concluded experimentally [21]. However, the reason for the activity loss of Nars with the increase in tungstate concentration in the environment needs to be further investigated (Additional file 1).

## Additional file

**Additional file 1.** Supplementary material containing the Cartesian coordinates of all the optimized geometries.
