# Supplementary material for: Effect of molybdenum and tungsten on the reduction of nitrate in nitrate reductase, a DFT study
Source: Chem Cent J. 2017 Apr 26;11:35. doi: 10.1186/s13065-017-0263-7 (PMC5405038; doi:10.1186/s13065-017-0263-7)
Supplement: Supplementary file 1 — Additional file 1. Supplementary material containing the Cartesian coordinates of all the optimized geometries. [file 13065_2017_263_MOESM1_ESM.doc]

Supporting Information for

Effect of Molybdenum and Tungsten on the reduction of nitrate in Nitrate Reductase, a DFT study

By

Uzma Habib (Corresponding Author), RCMS, NUST, H-12 Sector, Islamabad,

Pakistan

**Characterization of transition structures**

Contribution of relevant internal coordinates to the eigenvector of the negative eigenvalue followed in the optimization procedure for model complexes.

|  | **Eigenvalue** | **R(M-O)** | **R(O-N)** |
| --- | --- | --- | --- |
| **5a** | -0.07223 | 0.81137 | -0.39895 |
| **5b** | -0.05804 | -0.41111 | 0.70486 |

Fig.1: Optimized geometries for the Mo (**a**) and W (**b**) containing active site model complexes **1**-**7**.


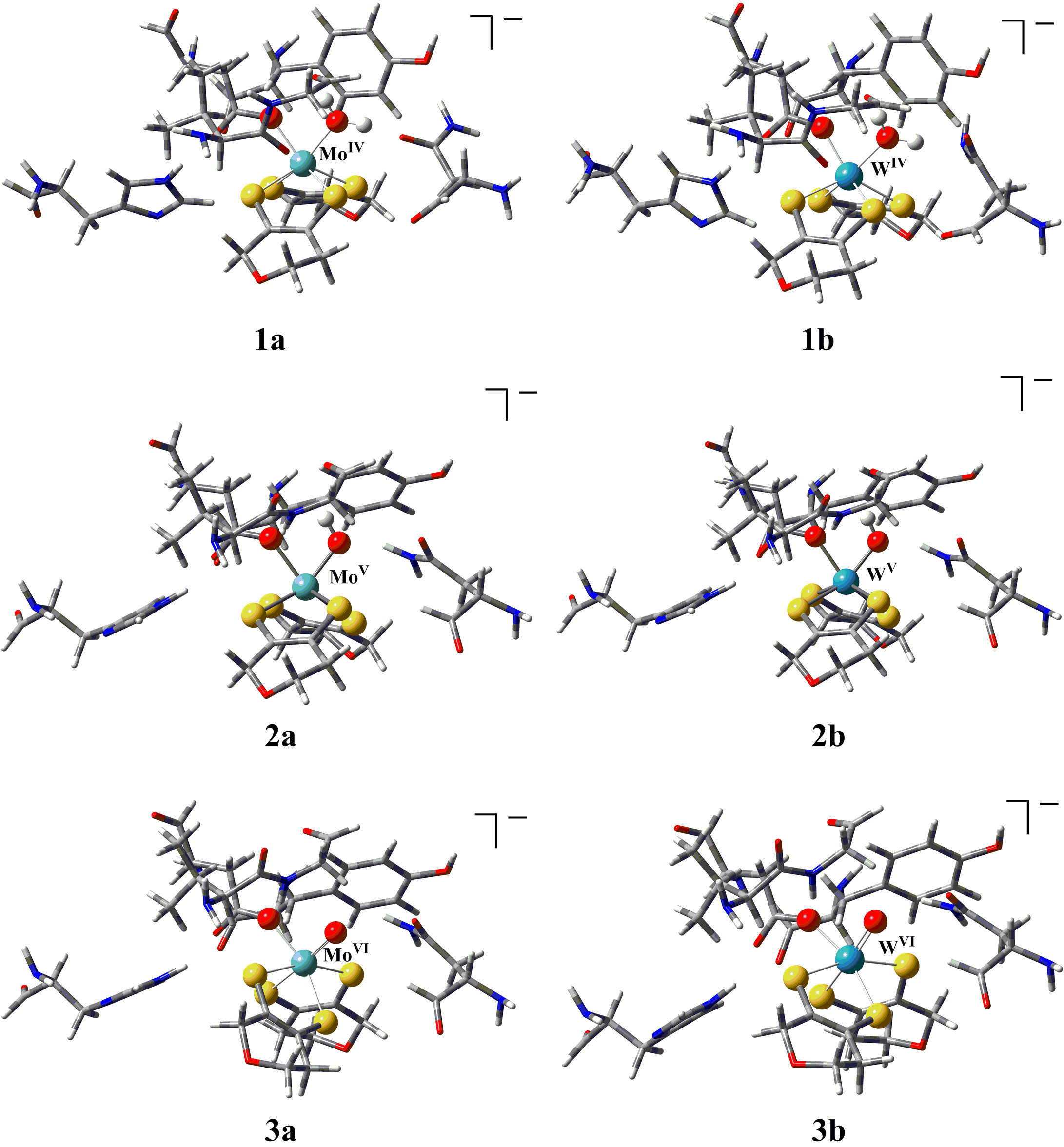


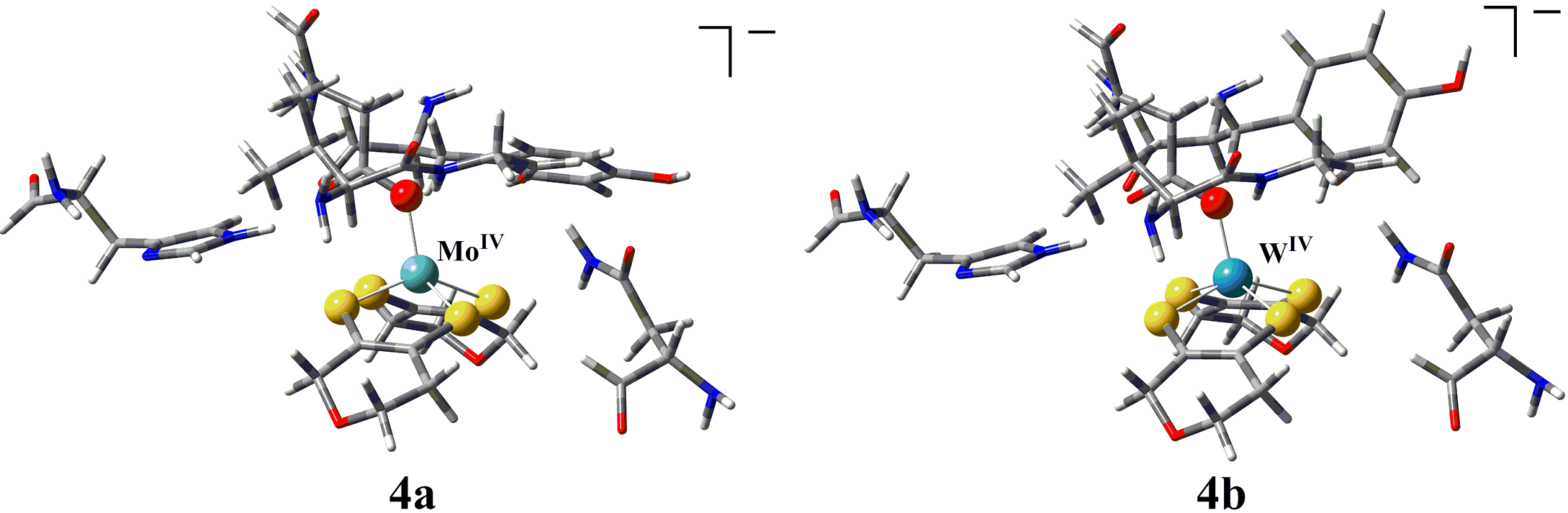


Fig.1. Continued…


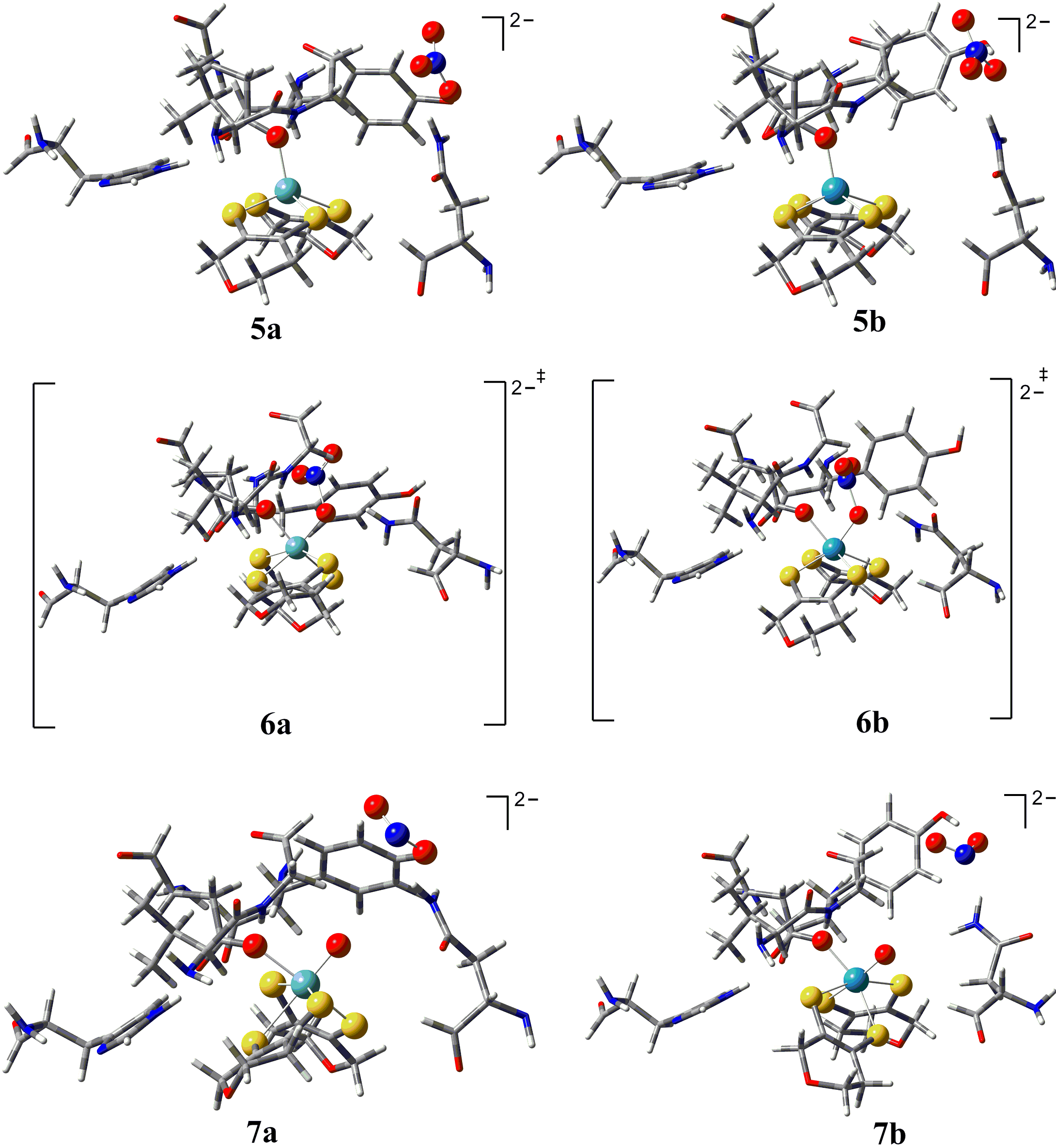


**Cartesian coordinates of structures optimized at B3LYP/Lanl2DZ* and electronic energies (*E* in a.u.) from single energy point calculations at B3LYP/SDDp**

**1a**, *E* = - 4693.8799274

N 8.94843500 0.86931100 -1.88627500

C 7.85758300 0.29820800 -1.08883600

C 7.33823100 -1.04398400 -1.62433400

O 7.93250100 -1.69555300 -2.50684000

C 6.72825100 1.33576600 -0.95046500

C 5.69023600 0.97441300 0.11720600

O 5.83375200 -0.02705100 0.88267300

N 4.62194700 1.81423600 0.17525700

N -3.00432100 5.58454200 1.31548600

C -2.61135300 4.35340200 0.60768300

C -3.84176800 3.45548600 0.42582300

O -4.11343000 2.81596100 -0.60780500

C -1.56692000 3.55808000 1.46656800

C -0.34114600 4.38422800 1.81999900

C 0.71580200 4.54775900 0.89199000

C -0.22365000 5.01045400 3.07954700

C 1.84148600 5.32432500 1.20205700

C 0.90100000 5.79346900 3.40582300

C 1.92933500 5.95142200 2.45885400

O 3.07842600 6.71958800 2.70874600

N -3.82027700 1.11689300 2.72103100

C -3.24009000 -0.17946600 3.11333400

C -3.57013200 -0.41494200 4.55406900

O -3.68724700 -1.53830600 5.08130300

C -1.71209800 -0.09439100 2.94093600

C -1.34523500 -0.24611200 1.46301900

O -2.20133500 -0.61719000 0.61881400

O -0.08450900 0.03675200 1.19148500

N -8.79193000 -2.60734700 -0.74843600

C -8.97825400 -1.21614400 -1.20318400

C -10.36179600 -0.97494800 -1.86517800

O -11.05253200 0.04973900 -1.70749300

C -7.88615500 -0.86716400 -2.28014000

C -6.48692900 -1.14935900 -1.80370300

N -6.04349300 -2.47878600 -1.66310900

C -5.46959700 -0.28110700 -1.41365500

C -4.78820300 -2.39695500 -1.19687900

N -4.40156500 -1.08987800 -1.03403400

N 0.66177400 -5.66713700 2.56789100

C 0.60912900 -4.24591300 2.20377600

C 1.95710500 -3.59460200 2.58961700

O 2.41666300 -3.61755100 3.77678800

C -0.63501100 -3.55281000 2.83587100

C -1.93952000 -4.03162800 2.14211600

C -0.73159900 -3.74802200 4.36724300

N 2.72949700 -3.07782700 1.58468000

C 4.09970100 -2.65560100 1.90050400

C 4.12670000 -1.72495400 3.10695100

O 3.48812900 -0.65036200 3.13303500

Mo 1.02336400 -0.06958400 -0.63877000

C 1.09509900 3.86707600 -3.48226500

O 0.07206900 4.18061300 -4.47209600

C 0.70922400 2.66530200 -2.64241900

S 1.97348800 1.84341800 -1.71982800

C -0.56103000 2.16575200 -2.64801500

S -0.93975100 0.70724100 -1.73669900

C -1.68648900 2.79080700 -3.45861600

C -1.27723500 4.20073600 -3.90898500

C 0.74307300 -4.57598800 -2.37905200

O 1.69602100 -5.19072800 -3.30143700

C 1.23339600 -3.24393600 -1.83146200

S 0.01165600 -2.17846000 -1.11286600

C 2.52851800 -2.83353200 -1.96533800

S 3.00031200 -1.20371800 -1.41843800

C 3.56580000 -3.69269300 -2.67343600

C 3.06759700 -5.14598600 -2.79323800

O 2.27045400 0.62819400 1.20835500

H 1.47865900 0.91960500 1.72023300

H 2.78150100 0.00006200 1.81651600

H -1.34177200 0.86716500 3.31243400

H -1.20370200 -0.88210500 3.50457200

H -3.68852000 0.51073500 5.15562500

H -4.83311600 1.15329800 2.85720800

H -3.61399700 1.30447700 1.73971000

H -3.60111000 -1.03105000 2.51826500

H 2.00573400 3.66772400 -4.05760700

H -1.89437100 2.16433100 -4.33749200

H -2.60740900 2.83001300 -2.86070800

H 1.27603500 4.75406300 -2.84606100

H -1.31376100 4.90526400 -3.05836800

H -1.92039100 4.57629000 -4.71093400

H 0.52428100 -5.28090300 -1.55595700

H -0.17472600 -4.43636700 -2.95917900

H 3.66238800 -5.71542400 -3.51414700

H 3.11523300 -5.64861800 -1.81067500

H 4.52531200 -3.67224200 -2.13591100

H 3.75459400 -3.28080400 -3.67506400

H -10.69287400 -1.81193900 -2.51569700

H -7.88481900 -3.01287700 -0.98541000

H -9.12869900 -2.81354400 0.18474800

H -8.88548900 -0.48148500 -0.38467100

H -7.99246700 0.18742500 -2.55876700

H -8.08449900 -1.47525300 -3.17441000

H -5.40529800 0.79534600 -1.37803500

H -4.14423400 -3.23211700 -0.97174500

H -4.50152700 3.43534300 1.31508400

H -2.25755600 6.01317900 1.85390100

H -3.53312100 6.25347800 0.76372600

H -2.17200200 4.53248300 -0.38865300

H -2.07040300 3.21727300 2.37926700

H -1.27227700 2.67532900 0.88717900

H 3.09543400 7.05948100 3.62654000

H 0.67150100 4.03607400 -0.06641800

H 2.65955200 5.44061400 0.49805000

H 0.97507600 6.26336800 4.38624600

H -1.00956700 4.87432900 3.82019100

H 0.90680600 -5.81920600 3.54360000

H 1.21359700 -6.24659600 1.94013000

H 0.49607300 -4.17513600 1.11539700

H -2.81338000 -3.57718900 2.62622600

H -1.94810000 -3.75165200 1.08098600

H -2.02386500 -5.12217800 2.22168100

H 0.18687000 -3.43535200 4.87593300

H -1.57715000 -3.17343400 4.76624700

H -0.91900800 -4.80363100 4.60647500

H -0.50568100 -2.48558600 2.62312900

H 2.37021600 -2.93808500 0.64303000

H 4.73039900 -3.52790900 2.11273500

H 4.50796300 -2.10131200 1.04718400

H 4.78278500 -1.99406600 3.94670400

H 4.48275400 2.51181600 -0.54559000

H 3.79109900 1.55948900 0.71993000

H 9.73849900 0.23169400 -1.98014000

H 8.63959200 1.16516500 -2.81299800

H 8.22488800 0.08715800 -0.07322700

H 7.19822400 2.29142000 -0.68527100

H 6.21646800 1.48189000 -1.91352500

H 6.41013400 -1.42077500 -1.16284300

H -3.50982200 -0.79128500 -0.63009000

**1b**, *E* = - 4692.776974

N 8.90423300 0.88332900 -1.82184100

C 7.80937800 0.31467800 -1.02857400

C 7.29528900 -1.03100800 -1.55998200

O 7.90049500 -1.69118000 -2.42840400

C 6.67650100 1.34952700 -0.90071300

C 5.63972000 0.98865000 0.16791600

O 5.79674100 0.00309300 0.95054700

N 4.55820200 1.81310100 0.20855700

N -3.05915300 5.59266300 1.34875800

C -2.66484600 4.36347100 0.63733100

C -3.89287300 3.46142000 0.46087300

O -4.16780100 2.81965200 -0.57036300

C -1.61470800 3.56936400 1.49059500

C -0.39045700 4.39833900 1.84273100

C 0.66536100 4.56352300 0.91367200

C -0.27365600 5.02569600 3.10183600

C 1.78937000 5.34310100 1.22255500

C 0.84935000 5.81164900 3.42676000

C 1.87652900 5.97142400 2.47878300

O 3.02397600 6.74258500 2.72750600

N -3.87376000 1.12650800 2.75991500

C -3.29898300 -0.17155700 3.15253300

C -3.62581200 -0.40211100 4.59589400

O -3.72151400 -1.52366600 5.13087700

C -1.77189600 -0.09568400 2.96656700

C -1.42104400 -0.26020300 1.48708300

O -2.27968400 -0.63700600 0.65330900

O -0.15724900 0.02348400 1.20283100

N -8.83512300 -2.60789200 -0.71333900

C -9.01830400 -1.22042400 -1.18144100

C -10.40429000 -0.97875100 -1.83580500

O -11.08789100 0.05179400 -1.68513000

C -7.93584900 -0.88732000 -2.27265600

C -6.53329400 -1.16610200 -1.80596600

N -6.08835000 -2.49428000 -1.65967800

C -5.51375400 -0.29441000 -1.42998200

C -4.82921300 -2.40847800 -1.20434100

N -4.44240600 -1.09997800 -1.05416700

N 0.61484100 -5.65359000 2.62653300

C 0.56036500 -4.23804800 2.24364300

C 1.91166100 -3.58344100 2.61000400

O 2.36889200 -3.56123000 3.79860700

C -0.67869400 -3.53651300 2.87503300

C -1.98711300 -4.02117500 2.19300100

C -0.76789100 -3.71509200 4.40895800

N 2.69119700 -3.11373900 1.58822700

C 4.06225200 -2.68675500 1.89437300

C 4.08712800 -1.73074200 3.08060100

O 3.45176300 -0.65345000 3.07977400

W 0.94963700 -0.07028000 -0.60476200

C 1.04096400 3.87602700 -3.42481400

O 0.02993700 4.18225100 -4.43048400

C 0.64457900 2.67841800 -2.58621000

S 1.91421100 1.83681100 -1.66940700

C -0.62174000 2.18193700 -2.60128200

S -1.00562100 0.70470300 -1.70399600

C -1.74265800 2.80691200 -3.41612700

C -1.32477900 4.21038400 -3.87999200

C 0.70498900 -4.58310600 -2.31205700

O 1.66021600 -5.18568600 -3.24150200

C 1.18697000 -3.24972500 -1.76396900

S -0.05638500 -2.17217300 -1.07548900

C 2.47658400 -2.83352100 -1.89278400

S 2.93282600 -1.18199100 -1.36464900

C 3.52409800 -3.68477200 -2.59354100

C 3.03112900 -5.13894700 -2.73099800

O 2.16768800 0.58586500 1.21507400

H 1.40859400 0.86751300 1.78047200

H 2.73108700 -0.03358800 1.79316000

H -1.39447500 0.86724800 3.32721000

H -1.26272900 -0.88144700 3.53205600

H -3.76380600 0.52534500 5.19040100

H -4.88494600 1.17134600 2.90527400

H -3.67510100 1.31125500 1.77651900

H -3.66869800 -1.02308900 2.56224900

H 1.95907100 3.67417700 -3.98759900

H -1.95517600 2.17272700 -4.28854200

H -2.66394900 2.85801100 -2.81928600

H 1.21264800 4.76833600 -2.79312000

H -1.36699700 4.92476000 -3.03770700

H -1.95990300 4.57867200 -4.69181300

H 0.49510300 -5.29475200 -1.49217900

H -0.21590800 -4.44874600 -2.88868800

H 3.62984900 -5.69809000 -3.45679500

H 3.07919400 -5.65222800 -1.75379600

H 4.47836900 -3.66792200 -2.04588500

H 3.72379500 -3.26371000 -3.58930400

H -10.74445900 -1.82053200 -2.47535200

H -7.93190700 -3.01979200 -0.95383300

H -9.16340400 -2.80165100 0.22552600

H -8.91461800 -0.47759800 -0.37174000

H -8.04302000 0.16398500 -2.56309200

H -8.14371000 -1.50594900 -3.15745300

H -5.45092500 0.78227200 -1.39924500

H -4.18257700 -3.24143300 -0.97884100

H -4.54837100 3.44059100 1.35345700

H -2.31003800 6.02447800 1.88147100

H -3.59304500 6.25963400 0.79939900

H -2.23012100 4.54511100 -0.36042400

H -2.11430900 3.22446300 2.40400900

H -1.31931100 2.68963800 0.90676900

H 3.03856900 7.08647900 3.64385300

H 0.62130100 4.05104700 -0.04427000

H 2.60646300 5.46099900 0.51770900

H 0.92297400 6.28269800 4.40668700

H -1.05893500 4.88848000 3.84297100

H 0.85804500 -5.79568500 3.60394000

H 1.16126700 -6.24379000 2.00439200

H 0.43975400 -4.18175200 1.15530300

H -2.85830900 -3.56179500 2.67723000

H -2.00116600 -3.75166800 1.12932300

H -2.07177700 -5.11081200 2.28393300

H 0.15310600 -3.39705000 4.90957700

H -1.61175900 -3.13673000 4.80601200

H -0.95472500 -4.76796500 4.66041800

H -0.54823800 -2.47211000 2.64970700

H 2.33707000 -3.01018600 0.63955300

H 4.69325800 -3.55419300 2.12481700

H 4.46818700 -2.15065500 1.02856000

H 4.74029900 -1.98019700 3.92786500

H 4.39969600 2.48660400 -0.53122000

H 3.73444800 1.55967600 0.75918200

H 9.69595500 0.24664300 -1.90733700

H 8.60124900 1.17509200 -2.75176200

H 8.17082600 0.10922700 -0.00974000

H 7.14207300 2.30820300 -0.63874700

H 6.16639600 1.48892100 -1.86553100

H 6.35986900 -1.40284500 -1.10886200

H -3.55021100 -0.79720600 -0.65670000

**2a**, *E* = - 4692.6764952

N 9.06411600 0.51758000 -1.75953200

C 8.00073100 -0.16198400 -1.01511400

C 7.33314700 -1.30573500 -1.79580900

O 7.69958200 -1.65519000 -2.93535000

C 6.97436500 0.89133000 -0.54604300

C 5.97942300 0.36730400 0.49241100

O 6.12309700 -0.75221600 1.06100700

N 4.93495500 1.20860600 0.76666800

N -2.57515800 5.42037800 2.22081200

C -2.28975600 4.37720300 1.22587900

C -3.54367500 3.51809100 1.00879500

O -3.92880900 3.09194500 -0.09652500

C -1.16566900 3.42555100 1.72404600

C 0.16974800 4.09002000 2.04956300

C 0.59317200 5.30396400 1.46207200

C 1.06475900 3.43019800 2.91981100

C 1.86369000 5.84292800 1.72635000

C 2.34103600 3.95212500 3.19019100

C 2.73846400 5.16117600 2.58861500

O 3.99939600 5.74005600 2.81233800

N -3.68277700 0.84769500 2.90273000

C -3.20102600 -0.53974900 3.06533700

C -3.53974100 -0.96906100 4.46737100

O -3.94330200 -2.10439600 4.78239000

C -1.67423800 -0.53849500 2.83761500

C -1.36997200 -0.41707800 1.34493100

O -2.29346700 -0.32967700 0.49254600

O -0.08381000 -0.40055600 1.04991600

N -8.86788500 -1.95058000 -1.08582300

C -8.95526600 -0.50097100 -1.34930600

C -10.32461400 -0.06403500 -1.91961600

O -10.90075400 1.00325900 -1.63431100

C -7.88168100 -0.09454400 -2.42312400

C -6.49435400 -0.50062500 -2.01811100

N -6.09762000 -1.84837800 -2.09833800

C -5.45982900 0.25552800 -1.47386000

C -4.84807100 -1.88796000 -1.60988600

N -4.42469500 -0.63969900 -1.22403300

N 0.35476500 -6.09895200 1.64666200

C 0.32920100 -4.65679100 1.90482500

C 1.53497300 -4.17691900 2.74517400

O 2.10305000 -4.91674000 3.60035200

C -1.02048700 -4.24159900 2.56980500

C -2.13413800 -4.25310400 1.50153400

C -1.38729100 -5.11935100 3.78620700

N 1.94275600 -2.88930000 2.49015300

C 3.06321000 -2.26460500 3.17084900

C 2.69814500 -1.07462300 4.03987300

O 1.55003600 -0.66554800 4.28721100

Mo 1.21638400 -0.08752700 -0.54788500

C 1.35224400 4.28734800 -2.70913600

O 0.43049700 4.76050800 -3.73418400

C 0.89323000 2.97961400 -2.07219900

S 1.97236400 2.19510900 -0.96691200

C -0.37941100 2.46307600 -2.31011900

S -0.87550200 0.93550500 -1.68341200

C -1.40120200 3.24551500 -3.12965300

C -0.96452500 4.70657700 -3.29761300

C 0.94465600 -4.40618500 -2.75659600

O 1.44370300 -4.76234600 -4.07813300

C 1.23278200 -2.95631600 -2.40364000

S 0.37657000 -2.31174400 -0.98980400

C 2.08585100 -2.18005700 -3.11643200

S 2.33723800 -0.46567700 -2.68849700

C 2.82182400 -2.71856900 -4.33483900

C 2.79718300 -4.25955900 -4.32233100

O 2.54950900 -0.15527800 0.59204700

H -1.20984600 0.30117500 3.36623800

H -1.19030800 -1.44022800 3.21772700

H -3.39829500 -0.17942600 5.23418500

H -4.67207500 0.95424400 3.13767000

H -3.53364800 1.14020600 1.93655000

H -3.65937100 -1.25362100 2.36459700

H 2.31126000 4.15176900 -3.21939800

H -1.50229600 2.77569700 -4.11706400

H -2.38126800 3.18661600 -2.63754700

H 1.47789200 5.06558300 -1.93565900

H -1.08201000 5.26187200 -2.35028000

H -1.53663000 5.21295100 -4.08028200

H 1.38695000 -5.07785500 -1.99834300

H -0.13416600 -4.59368700 -2.79010100

H 3.08656800 -4.67345100 -5.29292700

H 3.48157200 -4.64270600 -3.54676800

H 3.86395200 -2.36885400 -4.33936100

H 2.34716000 -2.34177500 -5.25221200

H -10.75871700 -0.79084700 -2.63828100

H -8.00188300 -2.38536000 -1.40633000

H -9.18339500 -2.24935900 -0.17023900

H -8.77135900 0.11138500 -0.44996400

H -7.93803000 0.98847000 -2.58048500

H -8.13858300 -0.59111400 -3.36930900

H -5.36720900 1.30588200 -1.24542900

H -4.23671400 -2.77191600 -1.51903600

H -4.10511500 3.32689900 1.94219300

H -1.82188400 5.64057300 2.86179600

H -3.10411800 6.22395900 1.90223800

H -1.99965400 4.78108200 0.23933000

H -1.53164900 2.89161400 2.61161700

H -1.00391300 2.67166200 0.94089600

H 4.55881100 5.17482000 3.38327700

H -0.06748300 5.84044900 0.78569800

H 2.19004300 6.77477200 1.27399000

H 3.01635200 3.41586100 3.85577600

H 0.77613400 2.48598500 3.37673100

H 0.57193600 -6.62874100 2.49088600

H 0.98966900 -6.35995200 0.89376200

H 0.37740000 -4.14290600 0.93844600

H -3.10649600 -4.00688100 1.94996600

H -1.93039000 -3.53147600 0.70007700

H -2.20133000 -5.25233100 1.05396000

H -0.56993400 -5.15641800 4.51811100

H -2.27755000 -4.71370000 4.28392300

H -1.61351500 -6.14395100 3.46531800

H -0.88802800 -3.21541600 2.93241100

H 1.46359000 -2.34209100 1.78057400

H 3.54060700 -3.02332600 3.80876200

H 3.82432300 -1.92078500 2.45211200

H 3.58542000 -0.55295600 4.45946400

H 4.79306700 2.04245500 0.20819000

H 4.09320300 0.79464700 1.17238000

H 9.84658500 -0.09715500 -1.98110300

H 8.72187300 0.94985900 -2.61834100

H 8.42329000 -0.62143500 -0.10986000

H 7.55039300 1.71786200 -0.11022200

H 6.42460300 1.30038100 -1.40650300

H 6.50838900 -1.82197400 -1.27416100

H -3.54338800 -0.43184000 -0.74694300

**2b**, *E* = - 4691.6003494

N 9.01874500 0.50245100 -1.72027800

C 7.97056500 -0.19075000 -0.96668500

C 7.28286100 -1.31618500 -1.75655500

O 7.62371000 -1.64205200 -2.91105600

C 6.96129500 0.85799900 -0.45202200

C 5.93516200 0.29983200 0.53773800

O 6.08598200 -0.82113400 1.10431100

N 4.85716700 1.10778400 0.76811900

N -2.60725400 5.43664700 2.26006800

C -2.33010600 4.40291600 1.25278300

C -3.58090300 3.53697700 1.04804400

O -3.96893500 3.09472400 -0.04991400

C -1.19258600 3.45516200 1.72987200

C 0.13567500 4.13231700 2.05707200

C 0.55571500 5.33891800 1.45191100

C 1.02698200 3.49505800 2.94719800

C 1.81904000 5.89247200 1.71898300

C 2.29650000 4.03189400 3.22108300

C 2.69025400 5.23318600 2.60231000

O 3.94376300 5.82695500 2.82909500

N -3.72720700 0.86697100 2.94198900

C -3.26247000 -0.52649100 3.10009800

C -3.58908100 -0.95016800 4.50662400

O -3.99453500 -2.08311000 4.82731700

C -1.73959100 -0.54427600 2.85053200

C -1.46294100 -0.47674400 1.35300800

O -2.38573800 -0.48787800 0.50396900

O -0.17481800 -0.39280400 1.04132300

N -8.91985200 -1.91730200 -1.04656900

C -8.99648700 -0.47104100 -1.32960000

C -10.37148500 -0.02683100 -1.88036200

O -10.93030300 1.05086700 -1.59990600

C -7.93995800 -0.08853700 -2.42927200

C -6.55215000 -0.51845200 -2.05270400

N -6.17666200 -1.87031600 -2.16025000

C -5.50051200 0.21325900 -1.50776000

C -4.92241200 -1.93676700 -1.68714000

N -4.47605600 -0.70144400 -1.28594300

N 0.29156800 -6.09055000 1.68591600

C 0.27082600 -4.64890900 1.94443300

C 1.47447600 -4.17286500 2.78902400

O 2.00474900 -4.89545000 3.68220700

C -1.07840000 -4.23147200 2.60926700

C -2.19234500 -4.23799100 1.54078800

C -1.44960900 -5.10938700 3.82441300

N 1.92172900 -2.90664100 2.49614800

C 3.02223300 -2.27089100 3.19853600

C 2.62326400 -1.07968000 4.05249900

O 1.46374000 -0.68984500 4.27595500

W 1.10331400 -0.06899800 -0.51523900

C 1.31684300 4.28242300 -2.67438600

O 0.39660900 4.76866600 -3.69493000

C 0.83774200 2.98210100 -2.04270100

S 1.92459800 2.17289100 -0.93549000

C -0.43082400 2.48944800 -2.26529500

S -0.95818200 0.97473300 -1.59035700

C -1.44855300 3.26564000 -3.09227700

C -1.00040100 4.72301300 -3.26432700

C 0.86235300 -4.40133800 -2.72609600

O 1.38410900 -4.75688800 -4.03887900

C 1.14414300 -2.95124700 -2.36955800

S 0.26987400 -2.30877200 -0.96508800

C 2.00774500 -2.17589800 -3.06769200

S 2.26251000 -0.46448900 -2.62473000

C 2.76627700 -2.71295500 -4.27210500

C 2.74187500 -4.25380600 -4.26007600

O 2.43129000 -0.15536600 0.64221200

H -1.25902600 0.30740200 3.34364700

H -1.25484000 -1.43648000 3.25245500

H -3.43729500 -0.15942100 5.27014200

H -4.71547000 0.98505100 3.17543700

H -3.57361800 1.17056100 1.98006100

H -3.73843800 -1.23516800 2.40630700

H 2.27074300 4.13558700 -3.19168600

H -1.55263300 2.79095100 -4.07713800

H -2.42975200 3.21803300 -2.60060400

H 1.45831800 5.05815300 -1.90059400

H -1.11893500 5.28090100 -2.31854700

H -1.56474000 5.23187300 -4.05123200

H 1.29117500 -5.07248400 -1.95971800

H -0.21552300 -4.58960800 -2.77890300

H 3.04768900 -4.66795100 -5.22545200

H 3.41286600 -4.63687300 -3.47293600

H 3.80819300 -2.36279400 -4.25721200

H 2.30888100 -2.33634000 -5.19818900

H -10.82711100 -0.75914500 -2.57986700

H -8.07530300 -2.37256600 -1.39329500

H -9.20936300 -2.19963300 -0.11754000

H -8.78926200 0.15053200 -0.44194500

H -7.98196200 0.99447600 -2.59071100

H -8.22504500 -0.58513600 -3.36723500

H -5.39078600 1.25814100 -1.26174800

H -4.32361500 -2.83108300 -1.61693700

H -4.13694600 3.35346800 1.98665800

H -1.84030600 5.66249800 2.88294700

H -3.14626000 6.23849100 1.95343800

H -2.05523700 4.81457300 0.26520900

H -1.54613200 2.90659000 2.61362200

H -1.02830600 2.71455800 0.93439600

H 4.50223100 5.27617500 3.41486000

H -0.10214400 5.85745800 0.75892600

H 2.14287900 6.81840200 1.25293200

H 2.96980200 3.51267100 3.90204900

H 0.74035400 2.55713900 3.41811400

H 0.49002800 -6.62341900 2.53266000

H 0.93435800 -6.35640900 0.94150900

H 0.32079600 -4.13555400 0.97806300

H -3.16356100 -3.98834500 1.98976000

H -1.98786600 -3.51778800 0.73890900

H -2.26344000 -5.23729200 1.09395100

H -0.63157300 -5.15343500 4.55484900

H -2.33674600 -4.69860800 4.32354200

H -1.68400100 -6.13168600 3.50183300

H -0.94172400 -3.20661600 2.97407400

H 1.47296900 -2.37566100 1.75577800

H 3.48879000 -3.02139800 3.85394200

H 3.79752900 -1.92681500 2.49601600

H 3.49325900 -0.54117300 4.48626800

H 4.72201200 1.94543800 0.21381900

H 4.00491900 0.67768800 1.13962800

H 9.79018500 -0.11084500 -1.98094300

H 8.65628200 0.96246500 -2.55596600

H 8.41169100 -0.67155400 -0.08147800

H 7.54906200 1.64378300 0.03985200

H 6.43949000 1.32856400 -1.29811000

H 6.47006200 -1.84354500 -1.22770200

H -3.59195500 -0.51708600 -0.80729500

**3a**, *E* = - 4617.4220479

N 9.07101100 0.83441900 -1.75707500

C 7.95146500 0.16845600 -1.07868500

C 7.38212300 -1.02577000 -1.85412900

O 7.89618100 -1.47961400 -2.89468800

C 6.83082600 1.17905700 -0.72862000

C 5.96602200 0.72266300 0.45520300

O 6.40993400 -0.08663800 1.32713300

N 4.72392500 1.27632500 0.53681000

N -2.62665600 5.28153900 2.57200000

C -2.38756000 4.38387300 1.42969000

C -3.56643000 3.41227100 1.28807900

O -4.02149400 2.99265400 0.20728200

C -1.11811300 3.51552800 1.65441900

C 0.19044100 4.28085600 1.83850900

C 0.37998900 5.61684000 1.41948300

C 1.29752900 3.59956400 2.39056200

C 1.62624400 6.25542900 1.54586800

C 2.55055600 4.22009700 2.51948700

C 2.71109100 5.55307100 2.09518700

O 3.93529300 6.23643000 2.19556000

N -3.62006500 0.65844100 3.06310700

C -3.00017400 -0.66096600 3.22369900

C -3.41551100 -1.22170900 4.54379400

O -2.72821700 -1.96327200 5.26926200

C -1.48654800 -0.57552400 3.00133000

C -1.23491000 -0.48992500 1.50468300

O -2.19324700 -0.55105200 0.69325400

O 0.03761900 -0.34692100 1.12290000

N -8.78950800 -2.08249900 -0.98518200

C -8.92050800 -0.62283300 -1.15364700

C -10.29968200 -0.19583100 -1.71742100

O -10.93882800 0.80481600 -1.34021200

C -7.83939900 -0.11603900 -2.17584900

C -6.44673600 -0.53589600 -1.79744500

N -6.04526000 -1.87725400 -1.94885300

C -5.40117500 0.20013500 -1.24661500

C -4.78353000 -1.93260200 -1.49589900

N -4.35658500 -0.70127900 -1.06368900

N 0.55997100 -6.12909900 1.45308200

C 0.54010300 -4.68498900 1.73013500

C 1.77247300 -4.29121300 2.58452800

O 2.36696200 -5.14043000 3.31311100

C -0.79714200 -4.26054400 2.42424700

C -1.97236900 -4.33773400 1.41897000

C -1.08513900 -5.09283000 3.69302800

N 2.22098600 -2.99537200 2.51432900

C 3.45733000 -2.63272800 3.19563600

C 3.87462800 -1.20479000 2.95652000

O 3.15991000 -0.35950600 2.38044200

Mo 0.72448000 -0.11618400 -0.75950300

C 1.24361800 4.36938900 -2.47502800

O 0.31778000 4.95589500 -3.44095400

C 0.73363900 3.03072300 -1.98393800

S 1.90469500 1.87290700 -1.29884000

C -0.56200000 2.65930700 -2.17100100

S -1.06991200 1.01236400 -1.76182500

C -1.60396800 3.55589800 -2.82016500

C -1.05571100 4.98591900 -2.94598300

C 0.48898800 -4.16936900 -3.28014700

O 1.54533800 -4.51603400 -4.21901800

C 0.94074700 -3.04084100 -2.37626600

S -0.32175200 -1.94472600 -1.79426900

C 2.25952400 -2.76362300 -2.17835300

S 2.72911300 -1.25891000 -1.33910600

C 3.36715300 -3.58486800 -2.82066300

C 2.78953000 -4.83366300 -3.51997500

H -1.08739100 0.31828800 3.49286500

H -0.96549600 -1.43899400 3.42552700

H -4.45642600 -0.96447800 4.84405100

H -4.48847300 0.78706300 3.58205500

H -3.74138900 0.89139000 2.07894100

H -3.39920800 -1.38192300 2.48134500

H 2.19103100 4.25448900 -3.01204600

H -1.85219700 3.15936400 -3.81472200

H -2.53119500 3.55046900 -2.22913000

H 1.39961600 5.06998600 -1.63556400

H -1.08557200 5.50078800 -1.96880600

H -1.61875100 5.57476700 -3.67676200

H 0.19476800 -5.06529600 -2.70310200

H -0.36332400 -3.85556000 -3.89030100

H 3.47193000 -5.21180100 -4.28713700

H 2.59879800 -5.63378100 -2.78257000

H 4.11128600 -3.89242500 -2.07060900

H 3.89398800 -2.95404100 -3.55048700

H -10.67958000 -0.86800200 -2.51563800

H -7.90911000 -2.47261000 -1.32454000

H -9.12226200 -2.45750400 -0.10489700

H -8.77496600 -0.06700300 -0.21132200

H -7.90704400 0.97547400 -2.24604300

H -8.08057100 -0.53529000 -3.16300900

H -5.30347700 1.24120400 -0.98110500

H -4.16075700 -2.81266000 -1.47099400

H -3.99371600 3.11632400 2.26320100

H -1.80913500 5.51978400 3.12191400

H -3.25946200 6.05764200 2.41273400

H -2.28121500 4.91727600 0.46840500

H -1.29024900 2.87490100 2.53083000

H -1.01609300 2.84876400 0.78733000

H 4.64866500 5.64933700 2.51937000

H -0.44461500 6.17254900 0.98091400

H 1.77293900 7.28087600 1.21979000

H 3.39229100 3.66437700 2.93084300

H 1.18958400 2.56119700 2.69645300

H 0.89595000 -6.65530100 2.25948700

H 1.08991500 -6.37137300 0.61845500

H 0.59722000 -4.14276200 0.77622600

H -2.92015000 -4.07836300 1.91137400

H -1.82362600 -3.65038700 0.57600800

H -2.05322000 -5.35866300 1.02686200

H -0.23664900 -5.08289000 4.38910800

H -1.95715400 -4.68754900 4.22146500

H -1.29668500 -6.13500800 3.42306600

H -0.66988000 -3.21383000 2.72885700

H 1.78044700 -2.29820400 1.91659300

H 3.37394100 -2.79407700 4.28397200

H 4.28422100 -3.28835600 2.87825300

H 4.88527100 -0.94081800 3.30825600

H 4.32097400 1.81417700 -0.22398800

H 4.08500300 0.93846100 1.25898600

H 9.89687000 0.24105200 -1.82390700

H 8.82047400 1.17393400 -2.68634100

H 8.30239100 -0.22870400 -0.11410500

H 7.33843800 2.10807000 -0.43851400

H 6.19893900 1.40683300 -1.59789400

H 6.46887300 -1.48318100 -1.42641300

H -3.45433900 -0.51032900 -0.62349200

**3b**, *E* = - 4616.3220931

N 9.04197600 0.72291600 -2.10616600

C 7.95435000 0.15380500 -1.29969400

C 7.39055000 -1.15702000 -1.86001700

O 7.88412000 -1.75970300 -2.83310900

C 6.82060900 1.18619100 -1.08188000

C 5.96841800 0.87744600 0.15807400

O 6.44389900 0.22524700 1.13901300

N 4.70528800 1.38282100 0.15616900

N -2.68538700 5.62590200 1.60635500

C -2.34975100 4.41959900 0.82744300

C -3.60370300 3.55010700 0.67177900

O -3.93438900 2.94390600 -0.36486800

C -1.27898600 3.56866700 1.59907700

C -0.03990600 4.36732000 1.96837400

C 1.01003900 4.55103400 1.03681100

C 0.09864000 4.94508300 3.24873300

C 2.14929900 5.30115000 1.36216800

C 1.23640600 5.70184500 3.59101400

C 2.25707900 5.88138400 2.63965300

O 3.41863800 6.62563000 2.90637400

N -3.57683900 1.13496900 2.88627200

C -2.94326400 -0.13886900 3.24932600

C -3.31377500 -0.46413800 4.65904000

O -2.59888900 -1.06893300 5.47828400

C -1.43732600 -0.08983500 2.96496300

C -1.24467500 -0.26091500 1.47035600

O -2.22073800 -0.49556100 0.71977100

O 0.01197400 -0.14208200 1.00716000

N -8.74279400 -2.34273900 -0.55507200

C -8.89964600 -0.94042100 -0.98631400

C -10.30115000 -0.63429700 -1.56949600

O -10.93774200 0.41698700 -1.36533800

C -7.86523600 -0.62342400 -2.12620000

C -6.45328900 -0.94460500 -1.72646500

N -6.02973700 -2.28446100 -1.63687200

C -5.40685800 -0.10302900 -1.35888400

C -4.75395800 -2.23563500 -1.22392600

N -4.33983900 -0.93902000 -1.04584600

N 0.71985100 -5.75059400 2.37370600

C 0.66354000 -4.28399200 2.44963000

C 1.88246300 -3.74602900 3.24020800

O 2.44559200 -4.44049500 4.13809000

C -0.68781400 -3.80800000 3.07914700

C -1.84901500 -4.03658200 2.08048300

C -0.97228700 -4.48472900 4.43808200

N 2.35168400 -2.49836000 2.91790200

C 3.57363800 -1.99917300 3.53593000

C 3.96740200 -0.64026200 3.01147700

O 3.23282800 0.04213600 2.27045900

W 0.64163400 -0.20649500 -0.86897200

C 1.11333800 3.96065400 -3.25547800

O 0.18400800 4.34267300 -4.31405800

C 0.60928400 2.73708900 -2.52036200

S 1.80493600 1.69698900 -1.67090000

C -0.68198600 2.33622700 -2.63650400

S -1.16551600 0.74265000 -2.00599100

C -1.73816900 3.10880500 -3.40704200

C -1.18456100 4.48141000 -3.82036100

C 0.51326200 -4.63257700 -2.65133700

O 1.59548500 -5.10097900 -3.50480100

C 0.93041300 -3.36446900 -1.94005800

S -0.35844200 -2.17801500 -1.61807400

C 2.23571800 -3.04123100 -1.75502800

S 2.66857300 -1.39087200 -1.18802000

C 3.37077600 -3.94179300 -2.21423500

C 2.82579200 -5.28921000 -2.73718300

H -1.01911500 0.87068500 3.28264900

H -0.89854000 -0.87104500 3.50909300

H -4.34678700 -0.16135800 4.94329500

H -4.45739600 1.31837400 3.36807900

H -3.69483900 1.21150400 1.87679200

H -3.36043300 -0.97727000 2.65512800

H 2.06138700 3.74746200 -3.76084300

H -2.02439900 2.53261400 -4.29819700

H -2.64238300 3.22592600 -2.79274700

H 1.26842900 4.81770600 -2.57312100

H -1.19884800 5.18085600 -2.96501200

H -1.75255900 4.91849100 -4.64741600

H 0.22559200 -5.43063300 -1.94246000

H -0.33253900 -4.44035900 -3.31864700

H 3.53276300 -5.76493800 -3.42362100

H 2.62611300 -5.97539500 -1.89465500

H 4.08393600 -4.12501500 -1.39623400

H 3.92706700 -3.42478600 -3.00903600

H -10.70055300 -1.44621000 -2.21330000

H -7.86732000 -2.78036500 -0.84576500

H -9.03950700 -2.54926900 0.39135800

H -8.72792400 -0.21787700 -0.16989700

H -7.95766800 0.43379200 -2.39943500

H -8.13179300 -1.22620600 -3.00591400

H -5.32230200 0.97070400 -1.29653000

H -4.11258100 -3.08571800 -1.05421300

H -4.22121500 3.51613600 1.59019900

H -1.90165000 6.02348600 2.11545300

H -3.23204400 6.32350400 1.10983700

H -1.95616000 4.63505200 -0.18082900

H -1.75720800 3.16426600 2.49889100

H -1.00639800 2.72634200 0.95318800

H 3.44406500 6.93756900 3.83385100

H 0.94769600 4.07165300 0.06311700

H 2.96346200 5.42967200 0.65575100

H 1.32654000 6.13481000 4.58708700

H -0.68058100 4.79030900 3.99269300

H 1.04370400 -6.15492800 3.25210000

H 1.27256600 -6.09341500 1.59067400

H 0.70857300 -3.87985000 1.42907400

H -2.80871500 -3.74137100 2.52760600

H -1.70721800 -3.45839700 1.15810000

H -1.90167500 -5.10019100 1.81863100

H -0.12916600 -4.37545600 5.13160800

H -1.85556000 -4.03247000 4.90666800

H -1.16595500 -5.55524500 4.29605700

H -0.58626400 -2.72963500 3.25723300

H 1.93573700 -1.93721400 2.17667400

H 3.47019000 -1.93987900 4.63232200

H 4.41208000 -2.69346900 3.36619600

H 4.96745200 -0.28098900 3.30226600

H 4.28697100 1.79059400 -0.67311400

H 4.08272500 1.15447600 0.93374300

H 9.87495900 0.13575200 -2.10942600

H 8.75777000 0.91530200 -3.06723000

H 8.33676600 -0.07928700 -0.29438900

H 7.31374300 2.15338400 -0.92059400

H 6.18322400 1.28495700 -1.97132600

H 6.50158400 -1.55850300 -1.33614800

H -3.43326100 -0.65614900 -0.67238100

**4a**, *E* = - 4897.8505582

N 8.46053800 0.04943800 -3.47020300

C 7.78924500 -0.71231700 -2.40112000

C 6.75526300 -1.71707400 -2.92840600

O 6.71609300 -2.07957000 -4.12242100

C 7.18851100 0.23161100 -1.33793200

C 6.99943500 -0.46884800 0.02006700

O 7.33003500 -1.68211400 0.18546000

N 6.48216500 0.30323600 1.01035100

N -2.49768800 5.80734100 1.23266700

C -2.32466100 4.55280800 0.47292600

C -3.62153900 3.74022000 0.53288200

O -4.15420200 3.15304700 -0.42966200

C -1.23264700 3.65401700 1.12294100

C 0.17974700 4.22163800 1.20346200

C 0.79680400 4.90446400 0.13117700

C 0.97536500 3.90387100 2.32658800

C 2.16928300 5.20430700 0.15373000

C 2.34975800 4.18709500 2.36288300

C 2.96381600 4.80541600 1.25009000

O 4.33183300 5.03110200 1.19441700

N -3.46235000 1.45001600 2.87107200

C -2.91486300 0.11494000 3.21517600

C -3.07833900 -0.06395400 4.69578000

O -3.60883400 -1.05546200 5.23462500

C -1.42371500 0.09207600 2.80884200

C -1.33754500 -0.17328700 1.31369000

O -2.37677300 -0.37687500 0.63743000

O -0.11298100 -0.16386200 0.77156200

N -9.15977500 -1.90654100 0.24477900

C -9.29867800 -0.51129500 -0.22429300

C -10.73593900 -0.16877000 -0.68912900

O -11.32025100 0.90999300 -0.46815400

C -8.35705800 -0.27497800 -1.46295000

C -6.93345200 -0.66814000 -1.18491900

N -6.58429800 -2.02944900 -1.09126800

C -5.80945400 0.11603300 -0.93688200

C -5.27528000 -2.04734300 -0.79215000

N -4.77128200 -0.77583200 -0.69140600

N 0.39214400 -5.68754000 2.29361100

C 0.39853500 -4.21523600 2.26034000

C 1.69655900 -3.60965000 2.84580200

O 2.41417000 -4.24472700 3.67263300

C -0.83380900 -3.63992300 3.03071800

C -2.10319200 -3.84769500 2.18795100

C -0.98213100 -4.24198400 4.44493000

N 1.97806300 -2.32955900 2.44012100

C 3.05471500 -1.53612200 3.01514400

C 2.58890800 -0.50136800 4.03494200

O 1.40556100 -0.36316800 4.40348700

Mo 0.28983500 -0.31191600 -1.21168900

C 0.64676000 3.63473600 -3.94585000

O -0.39777100 4.04585500 -4.88443100

C 0.18088500 2.47955800 -3.08412200

S 1.42172200 1.44594700 -2.32907100

C -1.13971500 2.16228200 -2.99323300

S -1.61979000 0.68135600 -2.15226000

C -2.24219600 2.95376500 -3.67815700

C -1.67645900 4.27666000 -4.21665900

C -0.31753900 -4.79420300 -2.74032500

O 0.61694300 -5.40589900 -3.67428800

C 0.25745200 -3.51109600 -2.18023500

S -0.91238300 -2.26347700 -1.72180600

C 1.59520400 -3.25632200 -2.18973800

S 2.18415800 -1.62389100 -1.76748900

C 2.60612200 -4.25034100 -2.74014100

C 1.92925200 -5.60037200 -3.05598000

O 5.42908300 1.07339500 3.62415900

H -0.95132000 1.05503900 3.03372400

H -0.83485700 -0.65131800 3.35303700

H -2.69606800 0.77885300 5.30684700

H -4.45931800 1.52812900 3.08388500

H -3.32019900 1.62796400 1.87720600

H -3.43162900 -0.71129600 2.70866500

H 1.49868200 3.33953500 -4.56702800

H -2.65643200 2.35786900 -4.50431400

H -3.06292300 3.14583100 -2.97224000

H 0.95070200 4.50013800 -3.32877500

H -1.53776200 5.00139300 -3.39360100

H -2.32880300 4.72117100 -4.97584000

H -0.56671000 -5.51499200 -1.93945700

H -1.22555600 -4.58938900 -3.31612800

H 2.51275100 -6.17960100 -3.77877900

H 1.80945500 -6.19418300 -2.13194800

H 3.42656300 -4.40702400 -2.02397100

H 3.05629200 -3.82865000 -3.64987600

H -11.21845800 -0.98723300 -1.26517400

H -8.33358600 -2.38568600 -0.12141400

H -9.32554900 -2.04551400 1.23542300

H -9.02414500 0.22756400 0.54739100

H -8.41739200 0.78024800 -1.75283900

H -8.73625400 -0.87963000 -2.29947500

H -5.65313100 1.18381900 -0.91967600

H -4.67288600 -2.92995400 -0.64811500

H -4.07951200 3.73717400 1.54106800

H -1.66341000 6.09815500 1.73548800

H -2.94549100 6.56465700 0.72615800

H -2.07375000 4.71371400 -0.58845800

H -1.57130100 3.38566000 2.13249300

H -1.19723300 2.72671400 0.53240700

H 4.84460900 4.34397200 1.71805400

H 0.21769800 5.15829600 -0.75549500

H 2.65532700 5.68787300 -0.68928200

H 2.95136900 3.91449800 3.22656800

H 0.52332700 3.39030900 3.17420000

H 0.81521000 -6.02705300 3.15902000

H 0.87089300 -6.09591600 1.49148500

H 0.30915700 -3.89329100 1.21395400

H -2.99084000 -3.47961700 2.72128000

H -2.03818900 -3.31850900 1.22816900

H -2.23944400 -4.91724200 1.98366500

H -0.06401100 -4.12130300 5.03407100

H -1.80051800 -3.74200300 4.97926700

H -1.21793200 -5.31189400 4.38067500

H -0.66000200 -2.56417800 3.14614000

H 1.36415500 -1.86948800 1.77242400

H 3.76270300 -2.21209600 3.51341500

H 3.61671700 -1.00540600 2.23366500

H 3.39640400 0.14478100 4.42281400

H 6.22269600 1.28404800 0.91847900

H 6.33795300 -0.06535900 1.94560000

H 8.76475600 -0.56243400 -4.22852100

H 7.86288300 0.78576500 -3.84966300

H 8.53630000 -1.33036800 -1.87999200

H 7.88475800 1.07013900 -1.20654500

H 6.22872800 0.65473400 -1.66817600

H 6.06565500 -2.14338200 -2.17927700

H -3.81536300 -0.54204700 -0.40757700

N 5.27155300 2.35397400 3.41809600

O 4.81683400 3.13387100 4.34442800

O 5.57375000 2.85727100 2.22251100

**4b**, *E* = - 4896.7472936

N 8.43751600 -1.06093300 -3.59967600

C 7.74341600 -1.37257300 -2.33848100

C 6.76779900 -2.55156400 -2.45424000

O 6.76847400 -3.33563300 -3.42586500

C 7.05916600 -0.11390800 -1.76529300

C 6.86489500 -0.19721700 -0.23936600

O 7.15499400 -1.25014800 0.40179100

N 6.38697700 0.93363100 0.34294300

N -2.46811400 5.90059300 -0.77876700

C -2.24895700 4.44585400 -0.92400100

C -3.58412200 3.71502900 -0.71979800

O -4.04879400 2.82718100 -1.46147700

C -1.29156200 3.92989000 0.20182400

C 0.09519100 4.54982000 0.28934300

C 1.21752600 3.85154500 -0.21155100

C 0.32969300 5.74696500 1.00881000

C 2.52454100 4.30100300 0.03143100

C 1.63301300 6.21817800 1.24213000

C 2.74353000 5.47612200 0.78744100

O 4.01785400 5.91644000 1.10943700

N -3.33877600 2.33405800 2.24170300

C -2.78950700 1.17710700 2.99499900

C -2.89092800 1.51490500 4.45471000

O -3.51243400 0.83131800 5.29190700

C -1.32096900 0.98077500 2.56052000

C -1.31751500 0.25619400 1.22970900

O -2.37825700 -0.16449200 0.71410800

O -0.11871400 0.09841300 0.62957900

N -9.05929700 -1.72251700 1.03993800

C -9.22211500 -0.58341700 0.11116800

C -10.67922000 -0.39945200 -0.37486000

O -11.24408100 0.69969500 -0.53876900

C -8.35218900 -0.82223400 -1.17818600

C -6.91429400 -1.10574100 -0.85066400

N -6.54722400 -2.35308800 -0.31015400

C -5.79039300 -0.28763100 -0.93346900

C -5.22687000 -2.27158600 -0.07755200

N -4.73395600 -1.04457400 -0.44096400

N 0.58588200 -4.57700700 3.96487600

C 0.56840500 -3.16203800 3.56108600

C 1.82807000 -2.39064300 4.01529500

O 2.45938500 -2.70960300 5.06484800

C -0.70016100 -2.44438100 4.12968400

C -1.93333100 -2.88480700 3.32228700

C -0.89417600 -2.69004900 5.64214500

N 2.16509500 -1.31220800 3.23684200

C 3.15435800 -0.32007400 3.63868000

C 2.53827100 0.94973000 4.21854700

O 1.31449600 1.10287300 4.40972600

W 0.22096400 -0.70738200 -1.15373500

C 0.53680600 2.19100200 -4.98458500

O -0.50101900 2.21000700 -6.01723100

C 0.08433100 1.38277600 -3.78758800

S 1.34548500 0.65125700 -2.73549200

C -1.22619000 1.08155900 -3.60848900

S -1.68540200 -0.10711200 -2.36461300

C -2.34106500 1.59173800 -4.50434200

C -1.78937100 2.64352700 -5.47995600

C -0.28111300 -5.44713300 -1.04109600

O 0.65767100 -6.29699100 -1.76068800

C 0.26561400 -4.04187500 -0.94197000

S -0.93570700 -2.72800200 -0.97476400

C 1.59310400 -3.77708300 -1.04173300

S 2.14771200 -2.07446600 -1.21740500

C 2.62843000 -4.87293000 -1.23378200

C 1.97707400 -6.26955800 -1.12726800

O 5.47926000 1.82437200 2.90164800

H -0.81398900 1.94666900 2.45841000

H -0.72683600 0.42313200 3.29004000

H -2.38258600 2.45368100 4.75149800

H -4.32130800 2.50642800 2.46747500

H -3.25545800 2.15814900 1.24065800

H -3.34363900 0.24535700 2.81926700

H 1.41088600 1.72963500 -5.45629500

H -2.77039500 0.74640500 -5.06145300

H -3.14675800 2.02091400 -3.89150500

H 0.79755800 3.22823700 -4.70508000

H -1.66679400 3.61632000 -4.97049100

H -2.44196200 2.77598400 -6.34954000

H -0.48847100 -5.88280000 -0.04615600

H -1.20636400 -5.46064200 -1.62584400

H 2.57083100 -7.02644400 -1.64984200

H 1.87341200 -6.56294200 -0.06701800

H 3.43525600 -4.78501500 -0.49067500

H 3.09392300 -4.74415600 -2.22137500

H -11.19681400 -1.35946300 -0.58695300

H -8.25466700 -2.31653500 0.82711100

H -9.16685800 -1.49439200 2.02211000

H -8.90392600 0.37597400 0.55257300

H -8.43638100 0.05855000 -1.82491000

H -8.77149800 -1.68309700 -1.71851600

H -5.64667300 0.72180700 -1.28721900

H -4.60905800 -3.05229800 0.33638400

H -4.13993900 4.07466900 0.16940100

H -1.64665500 6.39625400 -0.44176400

H -2.88867500 6.34787100 -1.58899500

H -1.83800100 4.15445100 -1.90468800

H -1.80916500 4.06416800 1.15879200

H -1.17806200 2.85239400 0.03862600

H 4.63671100 5.14268800 1.29254800

H 1.07476400 2.92441400 -0.76343300

H 3.37638600 3.72527600 -0.31986800

H 1.81514400 7.11087000 1.83389500

H -0.51095900 6.27856100 1.45666800

H 0.96132100 -4.67681800 4.90919300

H 1.11028900 -5.16190500 3.31527900

H 0.50639600 -3.12040000 2.46523900

H -2.84651700 -2.41568100 3.71475100

H -1.84106200 -2.61588000 2.26193000

H -2.04621800 -3.97425400 3.38917700

H 0.00336600 -2.42025000 6.21237000

H -1.73199000 -2.08486700 6.01268000

H -1.12499800 -3.74627700 5.83098600

H -0.54597600 -1.36756500 3.98977600

H 1.62974000 -1.12751200 2.39328300

H 3.81032100 -0.76256200 4.39999000

H 3.80223400 -0.02806100 2.80267400

H 3.25978000 1.75260600 4.44938100

H 6.17217400 1.76744000 -0.19019000

H 6.19279600 1.00883700 1.34684500

H 8.83694300 -1.89874600 -4.02457800

H 7.81920000 -0.60665900 -4.27420700

H 8.48547100 -1.69274600 -1.59157900

H 7.70297200 0.74443000 -1.99686000

H 6.08542900 0.06617100 -2.24461400

H 6.08214100 -2.69576000 -1.60142100

H -3.77550300 -0.72369700 -0.28445000

O 4.55975200 3.74757600 3.65910000

O 5.43984000 3.66062400 1.56340900

N 5.15496000 3.08326000 2.72638300

**5a**, *E* = - 4897.7804224

N 9.06230400 1.25972600 -2.14657200

C 7.96157500 0.53580600 -1.50110600

C 7.40371800 -0.62001000 -2.34084700

O 7.95683700 -1.03435400 -3.38132500

C 6.85529700 1.52310200 -1.07768200

C 6.02120600 0.96031900 0.08295000

O 6.56172900 0.21982600 0.95759600

N 4.71977300 1.33407900 0.09398300

N -2.63642700 5.20128300 2.64477400

C -2.53646300 4.55246600 1.32796300

C -3.56536800 3.40417800 1.25437300

O -4.30181700 3.15178300 0.28148700

C -1.09434500 3.99235400 1.15892200

C -0.00190800 5.05106000 1.29118700

C -0.11154100 6.33628000 0.70966600

C 1.19814000 4.72720500 1.96109300

C 0.93481700 7.27060900 0.78966300

C 2.25566800 5.65073100 2.04630300

C 2.12016900 6.92074500 1.45804300

O 3.14434300 7.89027200 1.51029900

N -3.54343300 0.53877400 2.84384100

C -3.01338300 -0.83745900 2.87137400

C -3.28295400 -1.43089200 4.19354200

O -3.76905500 -2.56412700 4.39613800

C -1.47951800 -0.82351500 2.75319500

C -1.09933000 -1.12406500 1.32468200

O -1.98175600 -1.31413300 0.44872900

O 0.20855500 -1.09774700 1.09964000

N -8.73137500 -2.02160200 -1.29792000

C -8.90463600 -0.55548100 -1.30119100

C -10.28498200 -0.11816000 -1.88061800

O -11.01095500 0.77565800 -1.40328700

C -7.77236000 0.09977700 -2.18150000

C -6.39469300 -0.40902500 -1.83779000

N -6.03150000 -1.72867300 -2.17634600

C -5.33222400 0.20374500 -1.17522900

C -4.78009600 -1.89199000 -1.71846900

N -4.32080400 -0.75251000 -1.10988300

N 0.72376200 -6.05178000 0.72467200

C 0.63501700 -4.68265000 1.25689300

C 1.77834500 -4.38219200 2.25180000

O 2.42638900 -5.30796900 2.83161900

C -0.75013500 -4.41646900 1.93201700

C -1.83869700 -4.30777700 0.84772400

C -1.10330500 -5.48044700 2.99274400

N 2.01658300 -3.05479200 2.46737400

C 3.00158700 -2.58056800 3.42094300

C 2.40768800 -1.99274600 4.69232300

O 1.20889700 -2.02896700 5.02329800

Mo 1.11531200 -0.25664400 -0.60002800

C 0.52318000 3.73300400 -3.31867100

O -0.78462800 4.04975400 -3.86833500

C 0.39940100 2.58156000 -2.35057500

S 1.85214500 1.63577500 -1.98338800

C -0.82958500 2.25098000 -1.86320700

S -1.02659400 0.84473400 -0.85743500

C -2.13464800 2.93399500 -2.24876600

C -1.81453200 4.28236000 -2.87497200

C 0.32046400 -4.38461400 -2.95259100

O 1.26772300 -5.30539700 -3.55416100

C 0.97452700 -3.13284000 -2.40684400

S -0.15106700 -1.90622600 -1.89986400

C 2.32772000 -2.99521300 -2.25386900

S 2.97326900 -1.55625700 -1.44542800

C 3.27851800 -4.07063900 -2.75965100

C 2.53496300 -5.41947900 -2.83635200

O 2.33383100 0.32144100 0.76438700

H -1.06774700 0.15788000 3.02030400

H -0.98484700 -1.53979800 3.42145700

H -3.00275900 -0.77760200 5.04607400

H -4.53811700 0.57434700 2.61783000

H -3.01228500 1.10107500 2.18301900

H -3.42467900 -1.47766300 2.08430400

H 1.14582300 3.46626000 -4.17970400

H -2.66022900 2.30727600 -2.98351800

H -2.80453800 3.03721600 -1.39117800

H 0.95790100 4.62786900 -2.83559200

H -1.46248500 5.00818300 -2.11932200

H -2.66494600 4.71079200 -3.41657200

H -0.22884200 -4.89392400 -2.14197800

H -0.39495400 -4.14259500 -3.74633000

H 3.11013800 -6.16572100 -3.39544400

H 2.35816100 -5.80152800 -1.81611700

H 4.14555100 -4.17018100 -2.09116400

H 3.66306900 -3.80366300 -3.75553600

H -10.58256600 -0.69398000 -2.78332400

H -7.83905300 -2.34320700 -1.68204400

H -9.02870600 -2.49174300 -0.45073400

H -8.83458700 -0.12028200 -0.28999800

H -7.82204500 1.18871200 -2.06753500

H -7.97939400 -0.13937800 -3.23502200

H -5.20847000 1.19741900 -0.77164200

H -4.18610200 -2.78779700 -1.80234300

H -3.65471300 2.86702200 2.21408000

H -1.75630600 5.47460300 3.06529200

H -3.39328100 5.86109100 2.78015700

H -2.75968800 5.23619800 0.48999800

H -0.91937200 3.20421900 1.89958800

H -1.00723700 3.50756800 0.18264200

H 3.95360200 7.51906700 1.91740900

H -1.02034900 6.61628100 0.18061600

H 0.85401300 8.25465700 0.33660900

H 3.17736500 5.37042500 2.55603300

H 1.30822300 3.73714000 2.39603600

H 1.11239200 -6.68510500 1.42508000

H 1.25626100 -6.10691900 -0.14072500

H 0.72246800 -3.97622600 0.42259500

H -2.80217500 -4.02357400 1.29268300

H -1.58571700 -3.56695300 0.08702800

H -1.95800100 -5.28538300 0.35964400

H -0.33084700 -5.54863600 3.77031000

H -2.05437200 -5.22319300 3.47773100

H -1.20927800 -6.46516500 2.51938900

H -0.66818400 -3.45298900 2.44838700

H 1.46148100 -2.35403200 1.96817500

H 3.64612400 -3.42490400 3.70636700

H 3.63413800 -1.80304400 2.97337500

H 3.16028600 -1.50488700 5.34615700

H 4.30701100 1.84163300 -0.68241100

H 4.03316400 0.89344500 0.71731400

H 9.82440100 0.63681000 -2.41658900

H 8.74920900 1.77646500 -2.97029100

H 8.32244700 0.08642100 -0.56246000

H 7.36848900 2.42634800 -0.72362800

H 6.21434800 1.80799000 -1.92318100

H 6.47974600 -1.09573600 -1.96137700

H -3.40020100 -0.67272400 -0.66638100

N 1.85565900 0.69904700 2.37602600

O 0.88911700 1.51726600 2.52221000

O 2.77936000 0.53239900 3.23558300

**5b**, *E* = - 4896.7161867

N 8.97888400 0.99183700 -2.07753000

C 7.90718100 0.23088500 -1.41966200

C 7.26387600 -0.83038400 -2.32291000

O 7.77503900 -1.19923800 -3.40161400

C 6.86532800 1.19403100 -0.81850100

C 6.00270400 0.50138000 0.24845700

O 6.47814500 -0.44239300 0.94838600

N 4.74681500 0.99291100 0.37712500

N -2.62412200 5.19404100 2.72506300

C -2.32607700 4.25085600 1.62561700

C -3.59900400 3.45725600 1.29020300

O -4.05780300 3.28325400 0.14489100

C -1.19349100 3.27191600 2.09097200

C 0.06027800 4.02759100 2.50040200

C 0.96669900 4.50410200 1.52463200

C 0.35348100 4.27484000 3.85687100

C 2.11315100 5.22651800 1.88722500

C 1.50014300 4.99863200 4.23818500

C 2.37341800 5.47626700 3.24583300

O 3.53778700 6.21226900 3.55877000

N -3.67745600 0.55901600 2.81722200

C -3.26855300 -0.86552800 2.76192500

C -3.48786700 -1.44637900 4.12544200

O -4.19540600 -2.44265000 4.37637700

C -1.76237700 -0.94642400 2.47344800

C -1.46934400 -0.70120100 1.00055700

O -2.39717400 -0.63602700 0.15281900

O -0.18602500 -0.56260300 0.75326100

N -8.91391600 -1.74791300 -1.41147500

C -9.01588800 -0.27315300 -1.48320600

C -10.40333600 0.21535200 -1.96241600

O -11.00928300 1.20715600 -1.51074600

C -7.96924200 0.28692300 -2.51813000

C -6.57452700 -0.19778800 -2.24289100

N -6.21824400 -1.52450200 -2.55092200

C -5.49410900 0.44482300 -1.64145400

C -4.94605600 -1.65994700 -2.13733900

N -4.47340600 -0.49646000 -1.58809500

N 0.39670700 -6.11488300 0.58218800

C 0.26667900 -4.90116400 1.40539600

C 1.25033800 -4.94769500 2.59607500

O 1.58025100 -6.04674900 3.14034300

C -1.20399000 -4.70796600 1.90927100

C -2.11090600 -4.29450600 0.72706000

C -1.75176200 -5.96352900 2.62218300

N 1.74007900 -3.74592500 3.02208000

C 2.63778800 -3.63329800 4.15407800

C 1.96725100 -3.36339800 5.49390800

O 0.74784700 -3.30794900 5.71599000

W 1.00423000 0.08960000 -0.82183700

C 1.36094200 4.60985600 -2.69155600

O 0.27104300 5.35934000 -3.31828700

C 0.89301000 3.28980000 -2.11030000

S 2.13888800 2.11212800 -1.63458100

C -0.42861200 2.99811700 -1.98479200

S -0.94352300 1.41316000 -1.39576100

C -1.52856900 3.95615300 -2.41800400

C -0.95626200 5.37392300 -2.52548900

C 0.40743600 -3.60259700 -3.91241600

O 1.36225500 -4.02287700 -4.93477000

C 0.98480900 -2.54301600 -2.99470400

S -0.18458300 -1.63577600 -2.03042000

C 2.31549700 -2.26752100 -2.93985100

S 2.91585800 -0.99629800 -1.85293200

C 3.32042300 -3.00119700 -3.81523000

C 2.68860300 -4.28799800 -4.37698500

O 2.15328800 0.22195900 0.73797400

H -1.21299000 -0.21044000 3.07172500

H -1.36207200 -1.93062400 2.73829300

H -2.96736800 -0.90019800 4.93781900

H -4.68951800 0.66874900 2.89986000

H -3.34674200 1.04311900 1.98337100

H -3.81701900 -1.45236900 2.01270600

H 2.09432600 4.44890400 -3.49028700

H -1.92532600 3.64379200 -3.39539200

H -2.36756000 3.92551000 -1.70950200

H 1.83476000 5.23848000 -1.91374500

H -0.74769800 5.78573900 -1.52118300

H -1.63271300 6.05345500 -3.05608500

H 0.07584300 -4.48352100 -3.33223500

H -0.45455800 -3.21319600 -4.46470800

H 3.27513200 -4.69886800 -5.20642900

H 2.60814000 -5.04898700 -3.58004900

H 4.22385000 -3.25461200 -3.24025000

H 3.63922600 -2.34918900 -4.64199100

H -10.82741100 -0.40365400 -2.78208100

H -8.06238000 -2.12054200 -1.83846800

H -9.13791300 -2.14754500 -0.50669800

H -8.81587200 0.21338800 -0.51414900

H -8.01167800 1.38195500 -2.49751400

H -8.26957700 -0.04666900 -3.52196800

H -5.36204300 1.44673700 -1.26181900

H -4.35016700 -2.55551700 -2.21224800

H -4.12257000 3.08108000 2.19012400

H -1.81936100 5.40875900 3.30668000

H -3.15159800 6.01904700 2.45421800

H -1.99002500 4.75048200 0.70169000

H -1.57021300 2.67975000 2.93366800

H -0.96798400 2.58646000 1.26706900

H 3.72606400 6.17556500 4.51862000

H 0.79743200 4.26259800 0.47820300

H 2.82452500 5.56816900 1.14146300

H 1.71666600 5.16759200 5.29343800

H -0.29940200 3.86789400 4.62678700

H 0.61380400 -6.92634800 1.16111200

H 1.06477300 -6.01171700 -0.17855400

H 0.51061100 -4.03089700 0.78174700

H -3.13870500 -4.11475100 1.07213900

H -1.75034900 -3.38799700 0.22803300

H -2.12688200 -5.10078300 -0.01701200

H -1.10737400 -6.26765900 3.45601400

H -2.75594100 -5.76056400 3.01786100

H -1.82209100 -6.79866600 1.91336400

H -1.18140900 -3.89273700 2.64769200

H 1.52320000 -2.87126700 2.54481400

H 3.20791300 -4.56822500 4.26208000

H 3.35104000 -2.81809300 3.97381700

H 2.69978900 -3.21347500 6.32020000

H 4.38029700 1.68624600 -0.26816700

H 4.02421100 0.50914600 0.91417000

H 9.65108100 0.37729900 -2.53853500

H 8.61040200 1.65622100 -2.76027000

H 8.32438800 -0.32531300 -0.56542900

H 7.42758700 2.00464700 -0.33661600

H 6.23275000 1.64024400 -1.59789000

H 6.32706700 -1.28487700 -1.95271300

H -3.56836500 -0.39809000 -1.10982800

O 2.02802700 -0.95688700 2.89855900

O 1.15671000 1.11380100 2.82370400

N 1.58991700 0.06388700 2.26739400

**6a**, *E* = - 4897.8473885

N -8.45683000 -1.16382000 -3.50382500

C -7.90043700 -0.18842000 -2.54736400

C -6.84439100 0.73598500 -3.16789000

O -6.69645100 0.86002500 -4.40142200

C -7.38196200 -0.88562400 -1.27132700

C -7.34115800 0.07179300 -0.06639200

O -7.84387500 1.23630500 -0.13679700

N -6.76624300 -0.42350200 1.05762600

N 2.84672800 -5.83212000 1.57452500

C 2.55798900 -4.61785500 0.78225600

C 3.84925700 -3.78903500 0.65452600

O 4.36294900 -3.36444600 -0.39747500

C 1.53230200 -3.73834400 1.54093200

C 0.09734000 -4.24685000 1.66032400

C -0.52309200 -5.10059400 0.72322300

C -0.71835600 -3.66208000 2.65757400

C -1.91335500 -5.31905900 0.74841700

C -2.10571600 -3.85425000 2.68791300

C -2.72264000 -4.66061900 1.70038400

O -4.09282200 -4.82227300 1.63734500

N 3.59052800 -1.28743200 2.75512500

C 3.00868300 0.05978500 2.97145300

C 3.14408100 0.37949800 4.42579800

O 3.60910700 1.44345900 4.88338000

C 1.49925000 0.07169400 2.63255800

C 1.26350400 0.66147900 1.24701000

O 2.22665400 1.02849200 0.52824400

O -0.01834900 0.69964000 0.86975000

N 9.06935700 2.08575200 -0.28272700

C 9.28064300 0.65277100 -0.56296900

C 10.72765100 0.34900000 -1.06378900

O 11.41774600 -0.62320400 -0.70193900

C 8.26528000 0.18210500 -1.67397600

C 6.84479200 0.60321600 -1.38831000

N 6.48464300 1.96173300 -1.49747900

C 5.73439200 -0.13311000 -0.97709200

C 5.19006000 2.02685700 -1.15248100

N 4.69622700 0.78726700 -0.83280000

N -0.65171300 5.54188600 1.53077200

C -0.58422300 4.09384100 1.81284300

C -1.79271900 3.55304700 2.62598900

O -2.54206600 4.30359600 3.31878000

C 0.74768800 3.72243100 2.53699900

C 1.93859400 3.83551200 1.56929500

C 0.98531000 4.53219000 3.83316000

N -1.95495200 2.19754600 2.52501000

C -2.96107400 1.43622400 3.24702500

C -2.38142400 0.45805600 4.26234600

O -1.17371100 0.40269900 4.57502200

Mo -1.03329900 0.02260300 -0.87971500

C 0.24290300 -2.93724500 -4.52694600

O 1.58832900 -3.45763800 -4.74639100

C 0.16071200 -2.02236400 -3.32118500

S -1.42076300 -1.36093300 -2.89449600

C 1.26260900 -1.84615800 -2.53476600

S 1.10382800 -1.11068200 -0.96617000

C 2.63308100 -2.40325600 -2.86505400

C 2.41733400 -3.72606600 -3.58671900

C 0.13889400 4.52620400 -2.55987400

O -0.76412500 5.63506400 -2.84416300

C -0.57146200 3.21903700 -2.22361800

S 0.40940600 1.79144000 -2.18466200

C -1.91966300 3.20988800 -1.88952600

S -2.66754800 1.78717900 -1.22634500

C -2.77456700 4.46615800 -2.03204300

C -1.88847100 5.71945800 -1.91654400

O -2.29891200 -0.68907400 0.07437700

H 1.08958900 -0.94536000 2.65104000

H 0.89768700 0.62038500 3.36753700

H 2.78847600 -0.42444000 5.10144100

H 4.61097400 -1.27778200 2.75062900

H 3.23448100 -1.70878700 1.90144100

H 3.50323000 0.84354800 2.38465100

H -0.01126900 -2.42038100 -5.45971800

H 3.17623900 -1.71560100 -3.52840800

H 3.23172100 -2.54206200 -1.96512900

H -0.46794300 -3.77163600 -4.39305500

H 1.94307300 -4.46559700 -2.91875100

H 3.34359400 -4.14650000 -3.99177100

H 0.79514700 4.79257500 -1.71443900

H 0.75623900 4.41437500 -3.45670000

H -2.43556500 6.62222300 -2.20766800

H -1.50443900 5.83801400 -0.89406700

H -3.55838600 4.48627500 -1.26347700

H -3.27594700 4.46561700 -3.01069700

H 11.10740500 1.10091200 -1.78832000

H 8.21972700 2.48105900 -0.69111500

H 9.27349700 2.38144100 0.66454300

H 9.11771400 0.02110800 0.32669900

H 8.33363800 -0.90698200 -1.77549800

H 8.57448700 0.62992800 -2.62966900

H 5.59372200 -1.18927500 -0.80149500

H 4.58859400 2.92094500 -1.11755300

H 4.34884000 -3.63699400 1.63210300

H 2.08353900 -6.10599200 2.18721400

H 3.24758100 -6.60902000 1.05799200

H 2.18803600 -4.83261400 -0.23335100

H 1.93613300 -3.53542600 2.54248600

H 1.48017600 -2.77931300 1.00649900

H -4.60534900 -4.02470500 2.01151700

H 0.06385300 -5.56794100 -0.06642200

H -2.39780900 -5.94322800 0.00199600

H -2.72320300 -3.35828900 3.43266600

H -0.26584800 -3.00672700 3.40201800

H -0.51111700 6.08568900 2.38359200

H -1.56332300 5.79597500 1.14723900

H -0.58414300 3.56512600 0.85149100

H 2.85480800 3.44734000 2.03398100

H 1.76722500 3.27978100 0.64573400

H 2.10513400 4.88987100 1.30818600

H 0.11221000 4.49818200 4.49740500

H 1.84372900 4.11442700 4.37497500

H 1.21757300 5.58426800 3.61334400

H 0.64285700 2.67516500 2.83709400

H -1.31145400 1.66563100 1.93313000

H -3.62623400 2.13617500 3.77262200

H -3.58570600 0.85057300 2.55544300

H -3.11746000 -0.24278200 4.69608200

H -6.33689500 -1.35306200 1.16016700

H -6.70642800 0.16980600 1.87666800

H -8.68179900 -0.72186700 -4.39615700

H -7.82131900 -1.94707800 -3.66516600

H -8.70272900 0.48995500 -2.21781500

H -8.07502800 -1.70455800 -1.03740700

H -6.38993600 -1.33429100 -1.42079700

H -6.24073100 1.33165600 -2.45968800

H 3.74423200 0.63245900 -0.48413400

N -5.24595300 -1.73614100 3.23129700

O -5.44597000 -2.63574800 2.26253900

O -4.47646200 -2.11733900 4.19350100

**6b**, *E* = - 4896.8043245

N 8.63512400 -1.67454300 -2.23520300

C 7.52602600 -1.69328800 -1.25847000

C 6.79416000 -3.02729100 -1.18502600

O 7.22303000 -4.09867800 -1.65792300

C 6.50595000 -0.54472100 -1.50668100

C 6.90448700 0.70916300 -0.71702700

O 8.11084400 1.10441900 -0.72819500

N 5.90738300 1.30161500 -0.00579600

N -2.31216100 5.72439900 -1.36034600

C -2.12262600 4.26473600 -1.32472800

C -3.49381600 3.57524700 -1.25935000

O -3.86016500 2.61231200 -1.96432900

C -1.35187900 3.87174800 -0.01474200

C -0.07085100 4.63446500 0.28048500

C 1.18608700 4.14732000 -0.14468100

C -0.08508400 5.77896200 1.11468000

C 2.38300800 4.74568700 0.27249000

C 1.10513300 6.39445700 1.53544200

C 2.35722800 5.86123700 1.15028500

O 3.50022000 6.42846600 1.65558000

N -3.65076300 2.44774400 1.81353000

C -3.19076500 1.35272700 2.71177600

C -3.49908300 1.81185500 4.12465500

O -4.20507900 1.17606600 4.93081200

C -1.67438800 1.13014400 2.47827500

C -1.44727900 0.27448600 1.22892500

O -2.41133700 -0.16472200 0.55427100

O -0.16926600 0.04701000 0.90740200

N -9.30617400 -1.57876600 0.25533200

C -9.32934000 -0.49068000 -0.74565400

C -10.70176600 -0.34864100 -1.45212900

O -11.24222600 0.73431800 -1.74928700

C -8.27092100 -0.78714100 -1.87164700

C -6.89333000 -1.03698800 -1.32260400

N -6.61910600 -2.22678400 -0.62049800

C -5.75095000 -0.23983400 -1.34437100

C -5.33755500 -2.13202200 -0.23260200

N -4.77712300 -0.95137200 -0.65075100

N -0.17759600 -4.36951300 4.56968000

C -0.09366400 -3.02358500 3.98097500

C 1.18786500 -2.24573000 4.37579100

O 1.81637800 -2.47455800 5.45065900

C -1.36408400 -2.18664900 4.33515300

C -2.54742800 -2.68725400 3.48488900

C -1.69015300 -2.20991900 5.84422400

N 1.55845200 -1.28293600 3.47157600

C 2.64573600 -0.33095800 3.65965100

C 2.17508400 1.09980700 3.92407400

O 0.99801100 1.39795400 4.20656600

W 0.96168300 -0.70106400 -0.66451700

C 1.16976900 1.62043600 -4.94099900

O 0.15916400 1.55996300 -5.99513200

C 0.69058900 0.97862100 -3.64336600

S 1.85559900 0.79959500 -2.35144700

C -0.62658100 0.60839900 -3.46115700

S -1.15552700 -0.20541500 -2.02010900

C -1.68955000 0.91891100 -4.50852600

C -1.16690300 1.95236400 -5.51513500

C 0.19764800 -5.48238000 -0.03028400

O 0.53270400 -6.57386000 -0.94229900

C 0.59862600 -4.12107100 -0.57548400

S -0.05275100 -2.70972600 0.28454100

C 1.40133000 -3.96762600 -1.65573300

S 1.80680900 -2.34502400 -2.27991700

C 1.96068600 -5.16852000 -2.40509700

C 1.87063700 -6.42729000 -1.52141300

O 2.45384600 -0.31789600 0.17974700

H -1.15802300 2.08871700 2.35424100

H -1.17706200 0.65605000 3.32902600

H -3.06610600 2.79599000 4.39258900

H -4.66052000 2.59718100 1.87905900

H -3.40966200 2.22072300 0.84921200

H -3.71962400 0.40367200 2.54125100

H 2.03752800 1.08518000 -5.34018200

H -1.96318400 -0.00639600 -5.03377100

H -2.59124100 1.29230100 -4.00424600

H 1.46050600 2.67187700 -4.76964400

H -1.11504400 2.95293000 -5.05171900

H -1.79697900 2.00471800 -6.40868500

H 0.68063000 -5.65865000 0.94827100

H -0.88739100 -5.54363500 0.10954500

H 2.03240500 -7.34032800 -2.10346600

H 2.62065600 -6.37542700 -0.71410000

H 3.00948700 -4.99118700 -2.68330600

H 1.39807100 -5.32204000 -3.33751400

H -11.17726000 -1.32432000 -1.68946700

H -8.46746300 -2.16265800 0.21073900

H -9.58178900 -1.30570200 1.19232100

H -9.08677600 0.49459200 -0.31253900

H -8.25860400 0.05621900 -2.57155100

H -8.60216200 -1.67989700 -2.42183000

H -5.54289600 0.72837400 -1.77355000

H -4.79568400 -2.87045200 0.33669700

H -4.17371200 4.03834000 -0.51611700

H -1.56443800 6.23768700 -0.89937700

H -2.56113700 6.10747800 -2.26744000

H -1.57558900 3.86698300 -2.19606400

H -2.04011700 4.00602200 0.82624200

H -1.12843900 2.80155000 -0.09454500

H 4.31018800 5.78955900 1.61149500

H 1.23241200 3.25832300 -0.77155300

H 3.34076300 4.33829600 -0.03639500

H 1.09340300 7.24561300 2.21105500

H -1.03825900 6.15696600 1.48599400

H 0.11268900 -4.35870900 5.54881300

H 0.37485600 -5.05439600 4.05435800

H -0.08332400 -3.13804800 2.89151600

H -3.46926000 -2.14516400 3.73930900

H -2.35585500 -2.55263000 2.41295500

H -2.70951100 -3.75596300 3.67420300

H -0.82838400 -1.89235300 6.44556400

H -2.52630200 -1.53121100 6.05827100

H -1.98174500 -3.22075700 6.15699400

H -1.14695700 -1.14737800 4.06157200

H 1.01626900 -1.17770900 2.61831200

H 3.25924700 -0.65731200 4.51109000

H 3.28842000 -0.30605600 2.76910400

H 2.95950500 1.87233000 3.82762700

H 4.96820800 0.91503000 -0.01723500

H 6.05010100 2.13486600 0.58703200

H 9.19467700 -0.83044800 -2.13699500

H 9.16324500 -2.54246100 -2.27690300

H 7.89108600 -1.56081800 -0.21405500

H 6.51969100 -0.31874100 -2.57930100

H 5.48700600 -0.85503000 -1.24299000

H 5.84531300 -2.99567700 -0.61381200

H -3.83221100 -0.63501300 -0.41256600

O 4.60334400 3.27001600 2.74150200

O 5.46457400 4.77370100 1.34494700

N 5.44640300 3.52668400 1.79645500
